# Environmental determinants of hiking, jogging, and cycling along the Beijing-Tianjin-Hebei grand canal: implications for health promotion

**DOI:** 10.3389/fpubh.2026.1843018

**Published:** 2026-05-07

**Authors:** Zihan Cai, Yi Yu, Ming Lu

**Affiliations:** 1 School of Architecture and Art Design, Hebei University of Technology, Tianjin, China; 2 Center of Urban and Rural Renewal and Architectural Heritage Protection, Hebei University of Technology, Tianjin, China; 3 School of Architecture and Design, Harbin Institute of Technology, Harbin, China

**Keywords:** machine learning, multiscale spatial analysis, physical activity promotion mechanism, spatial patterns optimization, sustainable healthy regional planning, the grand canal of Beijing-Tianjin-Hebei

## Abstract

**Introduction:**

The Grand Canal in the Beijing-Tianjin-Hebei offers abundant ecological resources and socio-economic value that can support physical activity and social interaction, yet its use remains largely spontaneous and underutilized for regional health promotion.

**Methods:**

This study quantifies how canal-adjacent space promotes hiking, jogging, and cycling, identifies correlating features and spatial heterogeneity, and proposes optimization recommendations. Using participant movement trajectories, we combine XGBoost with multiscale geographically weighted regression to assess factor importance and spatial nonlinearity. K-Means clustering classifies canal-adjacent areas into ecology (52.9%), service (29.5%), living (11.7%), and mixed (5.9%) types; Natural Breaks delineates advantage zones for physical activity.

**Results:**

Promotion indices derived from factor weight coefficients quantifies conversion efficiency across spatial types. Illumination intensity and population density correlate strongly with three activity types. Hiking is mainly driven by slope gradient, accessibility to fitness facilities, and NDVI; jogging correlates with office complex, canopy height, and distance to bus station; cycling depends mostly on natural features.

**Discussion:**

Effects are generally nonlinear and spatially heterogeneous, centering on the Beijing-Tianjin segment with decreasing influence toward the periphery. In addition, comparing the four spatial types, mixed spaces show the highest conversion efficiency. We propose targeted development strategies and a progressive spatial enhancement model to strengthen sports functions.

## 1 Introduction

Worldwide, overweight and obesity, driven by dietary habits, social pressures, and insufficient physical activity, have become major public-health challenges. According to the World Health Organization, by 2022 about 2.5 billion adults were overweight and roughly 890 million were living with obesity. Obesity-related deaths and disabilities now exceed 120 million and have risen steadily over the past decade (1, 2). By 2021, China reported 402 million adults with obesity, representing approximately 28% of its population. Against this backdrop, the Global Action Plan on Physical Activity 2018–2030 recommends integrating physical-activity promotion into national policy, urban planning, and community initiatives. In 2024, the National Health Commission, together with 16 departments, issued an Implementation Plan for the “Weight Management Year” campaign. The National People’s Congress in 2025 further emphasized the central role of physical exercise in chronic-disease prevention and healthy living, elevating national fitness to a state strategy.

Urbanization and modernisation have shifted occupational structures and transport modes, increasing sedentary time at work and in daily life and reducing routine physical activity (3). This trend has become a major contributor to the high prevalence of obesity and chronic disease (4). Many countries have responded with policy measures and public campaigns: the US Department of Health and Human Services developed the “Move Your Way” initiative to encourage use of parks, trails, and waterfront access points (5); the National Institute for Public Health of Netherlands together with the Sports Council have prioritized waterfront footpaths and cycling networks in national infrastructure assessments (6); and the General Administration of Sport of China has promoted the “Walk the Grand Canal” fitness campaign, integrating long-distance activities such as brisk walking and jogging with linear heritage spaces to both advance public health and unlock the Grand Canal’s ecological and cultural potential. The Beijing-Hangzhou Grand Canal, a World Natural and Cultural Heritage site and an important component of China’s intangible cultural heritage, embodies substantial historical, cultural, and ecological value. By offering diverse opportunities for exercise, recreation, and social interaction (7–9), it strongly motivates healthy physical activity and contributes to improved cardiopulmonary function, circulation, and reduced anxiety (10–12).

The functional reconfiguration of waterside spaces can effectively boost residents’ engagement in physical activity. The Beeston Cut employs a deconstructed, modular design of its waterways; by leveraging relatively short channels it provides highly accessible waterside areas and, through integration of cycling, jogging, and historical interpretation, helps connect urban and rural zones (13). As one of the earliest examples of canal repurposing, the Amsterdam canal system gradually shed its commercial shipping role after industrialization and evolved into a multifunctional landscape combining commerce, ecology, and residential uses, offering sightseeing, waterside promenades, and boat tours (14). In summary, combining preservation of the Grand Canal’s cultural context with active-use programming, and systematically assessing canal-side suitability for varied physical pursuits, provides a clear pathway for linear heritage to move from “static conservation” to “dynamic revitalisation,” with practical benefits for public health, sustainable tourism, and local regeneration.

Existing research on the Beijing-Hangzhou Grand Canal has concentrated on cultural-heritage conservation, ecological restoration, and tourism development. Quantitative studies that treat the canal corridor as a public-health resource are scarce, and no systematic quantitative assessment or planning framework currently exists. Although previous work has indicated the canal’s potential to support physical activity (15, 16), and shown that linear activities tend to favor continuous pathways and favorable ecological conditions (17, 18), the Grand Canal basin spans urban centers, satellite towns, and rural peripheries, creating complex spatial typologies, large spatial scales, and frequent functional shifts between sections that generate variability and uncertainty in user behavior and preferences. However, few studies have quantitatively examined how such heterogeneity affects the canal’s capacity to promote physical activity. Accordingly, this study aims to identify and compare how different sections of the Grand Canal corridor are associated with different types of physical activity, and to translate these quantified results into differentiated planning strategies. In doing so, it seeks to provide a basis for identifying the health-promotion potential of canal-adjacent spaces and optimizing their spatial configuration. Using multi-source data and machine-learning methods, the study focuses on county-level spatial units along the Beijing–Tianjin–Hebei section of the Beijing–Hangzhou Grand Canal. It systematically analyses the effects that promote walking, jogging, and cycling, with the goal of quantifying the conversion efficiency of canal-side spaces in supporting physical activity, enhancing the value of spatial health and fitness resources, and providing quantitative evidence and planning recommendations for canal-based active-health promotion.

## 2 Literature review

### 2.1 The multidimensional impact pathways of the grand canal’s waterfront spaces on physical activity

Beijing-Hangzhou Grand Canal, one of the world’s six major canals, has been transitioning from a commercial shipping route toward urban recreation and cultural promotion (19, 20). Compared with urban green spaces and country parks, the Canal tends to more effectively encourage residents to engage in physical activity (21, 22). Research attention has progressively shifted from ecological and environmental issues toward human-centered topics, including cultural perception (23), therapeutic benefits (24), and physical activity (25). Based on the study subjects, these works can be categorized into three dimensions (Table 1).

**Table 1:** Dimensions of the grand canal’s impact on physical activity along its waterway.

Classification	Perspectives	Point of view
Service	Historical and cultural appreciation, commercial and entertainments	(1) Historical and cultural elements can significantly enhance residents’ willingness to travel and influence their behavioral preferences (115, 116).
		(2) Residents residing near entertainment and shopping centers typically exhibit higher travel willingness (117), though the effect varies across different types of physical activity (26, 30).
Living	Daily travel environment and infrastructure provision	(1) Highly accessible canal spaces are more likely to attract residents to visit (33, 118), while also providing conditions for low-cost jogging activities within the city (37).
		(2) Enhancing the quality and diversity of infrastructure within residential areas can similarly promote physical activity among residents (119).
Ecology	Ecosystem services, utilization of blue-green resources	(1) Individuals with varying levels of physical activity exhibit distinct preferences for ecological environments (61, 93), with ecosystems serving different physical activities through multiple pathways.
		(2) Ecological spaces integrating blue and green infrastructure more effectively encourage residents to engage in vigorous physical activity (56), with rivers demonstrating a stronger promotional effect than lakes (39).

Regarding cultural services, Sallis et al. (26), Qiao and Yeh (27) reported that commercial distinctiveness and historical ambience tend to attract tourists to canal-side activities, whereas residents prefer waterfront cultural plazas and waterside promenades for daily exercise (28). Xie et al. (29), drawing on perceived-value theory, confirmed the positive role of commercial services and cultural identity in promoting physical activity. Moreover, features such as large shopping centers (30), restaurants and bars (31), and retail outlets (26) exert varying influences on activities like hiking and cycling.

Regarding living amenities, urban parks and outdoor fitness facilities form key recreational spaces in residents’ daily lives (32), and their accessibility significantly promotes a range of health behaviors (33). Conversely, inadequate facilities are associated with sedentary behavior and lower overall activity levels (34, 35). Liu (36) found that fitness stations along the canal increased residents’ exercise participation, with facility diversity and accessibility particularly supporting low-threshold activities such as hiking and jogging (37).

Regarding ecological environments, an integrated blue–green system not only supports leisure and restoration but also creates conditions for higher-intensity exercise (38). Noted that rivers and lakes are important promoting factors of physical activity, and waterside parks in particular tend to attract jogging and cycling (39). Spaces along the canal combine cultural heritage, ecological services, and commercial functions, producing multifaceted attributes that better accommodate diverse exercise needs (40). However, work on the canal’s ecological environment has mainly addressed landscape quality (41), ecosystem-service functions (42, 43), and ecological-performance assessments (44, 45). Although regional development models coupling ecosystem services with social benefits have been explored (46), further quantitative research is needed to clarify how ecological characteristics can more effectively promote residents’ physical activity and health behaviors.

### 2.2 Differences in environmental preferences for physical activity

Preferences for hiking, jogging, and cycling are shaped by spatial environments and multiple factors (47, 48). Much of the literature has examined how elements of the urban built environment influence activity preferences. Hikers are attracted to strong place character, expansive natural views, and well-maintained footpaths, typically favoring trails with open space, water features, trees, and rest facilities, while showing relatively negative attitudes toward artificial surfaces such as asphalt and concrete (49–51).

Joggers’ preferences are influenced by street-level scenery and by the accessibility and service quality of water bodies and green spaces (52–55), and empirical studies find that areas with high jogging intensity are often close to water or green space (56). Riverside jogging routes enriched with greenery, flowers, and bridges increase psychological satisfaction and health benefits (57), whereas commercial noise and heavy traffic inhibit jogging (58). Joggers also tend to prefer smaller recreational water bodies and greater vegetation cover in their surroundings (59).

Cyclists favor routes with extensive water coverage (60), flat terrain, and low visual complexity (61), such as waterfront greenways on urban fringes or expansive rural landscapes (60, 62–64), which support long, high-intensity linear activity and distant vistas (65). A Rotterdam study similarly found that beyond the appeal of water, low traffic interference and simple visual environments increase cycling intensity (66). However, most studies consider single environment types in isolation and thus do not fully reveal how different activities are distributed or interact within multifunctional, composite spaces.

### 2.3 Machine learning based identification of nonlinear effects and spatial heterogeneity

In physical activity research, scholars increasingly adopt a nonlinear perspective because behavioral data are complex and stochastic, and environmental effects on activity typically manifest as demand variations across spatial units rather than simple linear aggregations. Machine learning approaches outperform traditional linear models for fitting high-dimensional data and complex relationships, reducing estimation bias and regionalisation errors (67). Many studies employ algorithms such as Random Forest and Support Vector Machines to analyze how the built environment shapes resident behavior, thereby validating nonlinear effects (68). To mitigate the “black-box” problem and account for localized effects, hybrid frameworks that combine machine learning with interpretable or spatially explicit methods have become mainstream. For example, Yang et al. (69) constructs an interpretable GBDT + SHAP pipeline that reveals an inverted-U threshold relationship between outdoor jogging and built-environment factors. Likewise, Zhang et al. (66) integrates machine learning with computer-vision techniques to extract street-level visual features, and work incorporating crowdsourced observations highlights how subjective visual environments influence jogging and cycling (60). Recent studies have combined interpretable machine learning with spatial heterogeneity analysis to identify differences in the spatial scale, direction, and strength of environmental effects. Using an ensemble framework based on RF-VI and MGWR, Zhang et al. (70) revealed the heterogeneous effects of natural and built-environment factors on outdoor jogging across multiple spatial scales. Other studies then further demonstrated that factors such as green-view ratio, openness, and walkability exert significant influences on jogging, with clear scale effects and spatiotemporal variation (71). These findings suggest that research on physical activity should focus not only on whether exercise occurs, but also on how it unfolds within specific spatial contexts.

Environmental effects often exhibit spatial heterogeneity, a pattern that is particularly pronounced in physical activity outcomes. Influence mechanisms differ markedly across areas, and hybrid approaches that combine spatial econometric models (e.g., GWR, GTWR) with machine learning can effectively reveal these variations. Lotfata and Georganos (72) introduced the Geographically Weighted Random Forest (GRF) in a study of Chicago, constructing local random forests for each spatial unit to identify how socio-ecological factors of community physical activity vary by context. Similarly, Li (73) compared XGBoost, Spatial Lag Model (SLM), and Multiscale Geographically Weighted Regression (MGWR), demonstrating that integrating machine learning with spatial-econometric techniques more effectively detects spatial heterogeneity and the behavioral mechanisms underlying human activity across locations. In summary, big data and ensemble machine learning approaches offer clear advantages for analyzing spatial environments and activity patterns (74), opening new avenues for more comprehensive and precise physical-activity research.

### 2.4 Research gap

The Grand Canal spaces possess clear advantages for promoting diverse physical activities because of their rich ecological resources and strong cultural potential. Although research on links between urban–rural environments and physical activity has gained prominence recently (75, 76), systematic quantitative studies that specifically address waterfront contexts remain limited. First, existing work typically focuses on single activity types and on isolated environment categories, which constrains the ability to reveal differences in spatial demand and response mechanisms across activities. Second, although many studies have begun to incorporate interpretable machine learning and spatial analysis methods, these approaches have been applied mainly to the general urban built environment (77), with insufficient attention to the complex spatial characteristics of linear heritage corridors that integrate waterfront environments, cultural nodes, service facilities, and urban–rural transition features. Finally, although some studies demonstrate promotional effects of spatial elements, these effects are rarely integrated and quantified into comparable spatial indices, limiting the translation of findings into actionable regional planning and spatial-enhancement strategies.

We first categorizes spatial factors along the canal into three dimensions—service, living, and ecology—using behavioral-trajectory big data and multi-source geographic datasets, while classifying physical activities into hiking, jogging, and cycling by pace and intensity. Secondly, leveraging multi-source big data, we establish an analytical framework that combines interpretable machine learning (XGBoost and SHAP) with MGWR to identify the key factors associated with different types of physical activity, as well as their nonlinear responses and spatial heterogeneity. We further employ K-means clustering and the Natural Breaks method to delineate physical-activity advantage zones, thereby calculating the physical-activity promotion index along the corridor and quantifying the spatial conversion efficiency of activity promotion. Finally, based on these results, we propose targeted spatial pattern optimization strategies and progressive enhancement models to improve the canal’s capacity for promoting physical activity.

This study uses the Grand Canal, focusing on the Beijing-Tianjin-Hebei section, as a case study with three specific objectives: (1) Identify the key promoting factors of hiking, jogging, and cycling along the canal space, and quantify their threshold effects and interactive influences. (2) Analyze scale-dependent spatial effects of these promoting factors, compute physical activity promotion indices, and quantify conversion efficiency for each activity across the defined spatial types. (3) Translate the findings into spatially targeted optimization recommendations and delineated advantage zones to guide planning and enhancement of the canal’s capacity to promote active health.

## 3 Materials and methods

### 3.1 Research design

**Figure 1:**
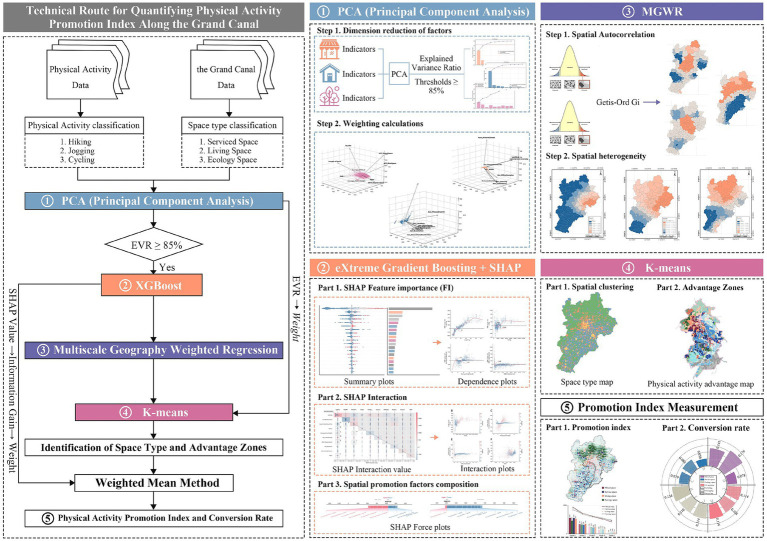
Technical route.

This study follows a four-stage logical framework: theoretical framework development, data collection and preprocessing, identification of functional characteristics, and formulation of planning insights along the Grand Canal corridor. Guided by social-ecological theory and a targeted literature review, we first identify candidate impact factors and organize them into three dimensions. The research comprises five principal steps (Figure 1): (1) Data preprocessing, including Pearson Correlation analysis and Principal Component Analysis (PCA), to construct a coherent set of influencing variables for the Grand Canal physical activity. (2) XGBoost regression for hiking, jogging, and cycling to screen key determinants, reveal action thresholds and interaction effects, and rank factor importance. (3) Building MGWR models for each activity using the selected key factors to characterize and compare scale-dependent spatial heterogeneity. (4) Applying K-means clustering to segment spatial types and using the Natural Breaks method to delineate activity advantage zones by overlaying cluster types with activity intensity maps. (5) Computing physical activity promotion indices via weighted aggregation of factor coefficients to quantify spatial promotion conversion efficiency along the Grand Canal corridor. This integrated technical route moves from promoting factor identification to spatial distribution mapping, providing an evidence base for targeted insights for Enhancing Planning and enhancement models for ecological wellness.

### 3.2 Study area

The Grand Canal section within the Beijing-Tianjin-Hebei encompasses the Northern, Southern, Wei, and Yongji canals, extending approximately 530 km across 58 counties. Spatially, the corridor follows a northwest-southeast gradient from mountainous terrain to plains. Its systematic spatial planning along the waterway traces back to 486 BC, and over roughly 2,500 years it has evolved into a multi-scale and composite functional system. The upper study area traverse core urban centers such as Beijing and Tianjin, while the lower area pass through counties including Qingxian and Botou before entering Shandong Province to Bo sea. Organized around the water network, the corridor has formed continuous greenways and composite landscape belts and, driven by the Healthy China policy, serves as an important conduit for health promoting behaviors.

**Figure 2:**
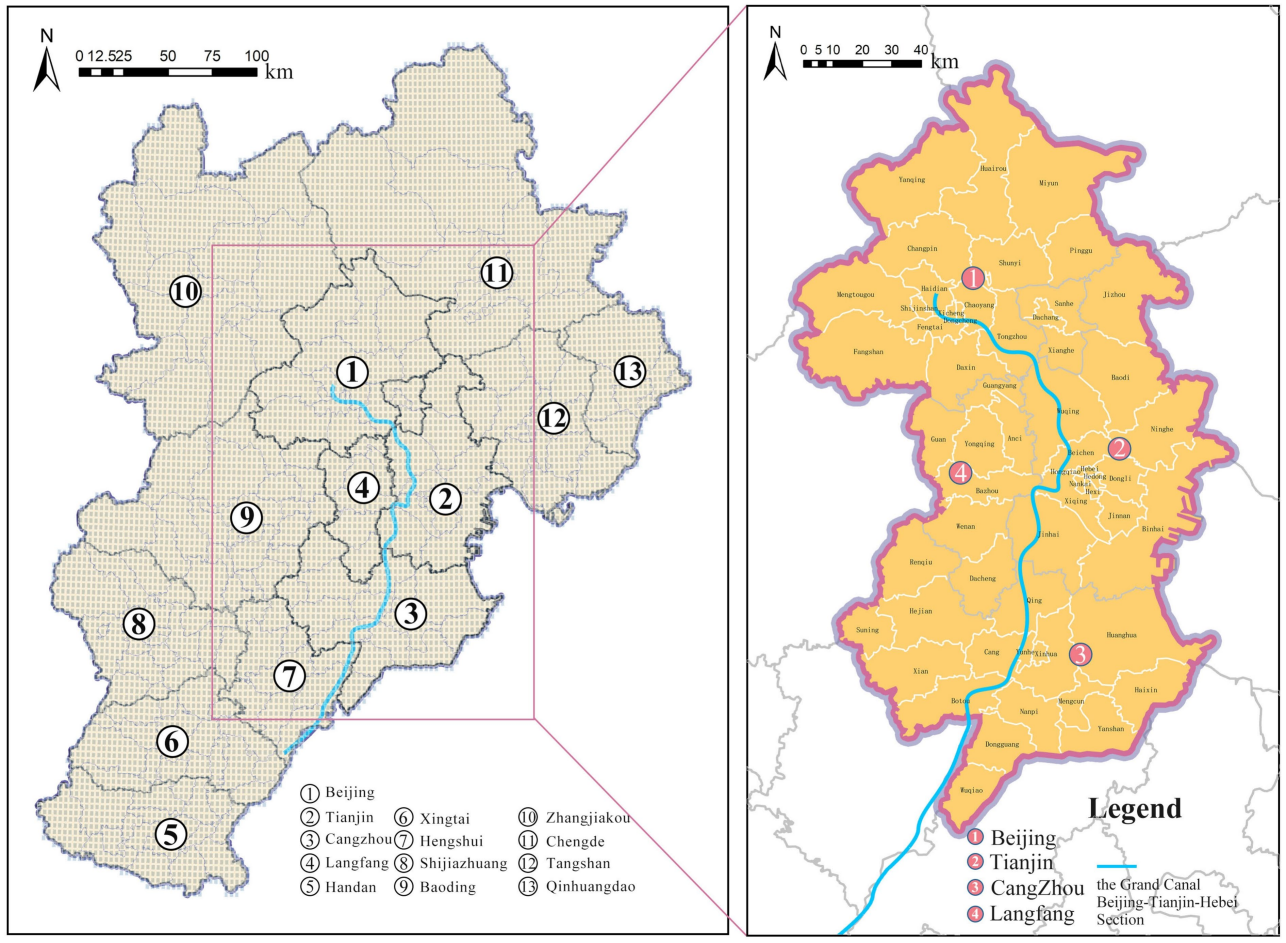
Study area.

Depending on the specific research objective and level of analysis, the empirical analyses in this study employ two spatial units: grid cells and county-level units. Grid cells are used primarily to characterize variations in the functional structure of canal-adjacent spaces and to identify physical-activity advantage zones, whereas county-level units are adopted to examine the regional spatial heterogeneity of socioeconomic attributes and enabling factors, thereby revealing intra-county dynamics within the broader regional context. Specifically, the study uses 2.5 × 2.5 km grid cells to generate 9,503 spatial analysis units. This choice is based on two main considerations. First, existing studies suggest that different physical activities have relatively stable duration ranges for health benefits, with common durations of 30–40 min for brisk walking, 20–30 min for jogging, and 30–60 min for cycling (78, 79). Based on average travel speeds, these durations correspond approximately to distance ranges of 2.1–7.5 km. Delineating 2.5 × 2.5 km grid cells therefore allows the study to better capture and compare the environmental conditions encountered within a single exercise session. Second, participants engaged in longer-distance activities, particularly cycling, often pass through multiple facility nodes and environmental segments in sequence, resulting in complex patterns of spatial exposure. This grid scale can comprehensively capture key environmental elements, including green space, water bodies, lighting conditions, public transport stops, and service facilities, while preserving spatial variability and improving comparability across spatial units (Figure 2).

### 3.3 Theoretical hypothesis, data acquisition and factor system construction

#### 3.3.1 Theoretical hypothesis

**Figure 3:**
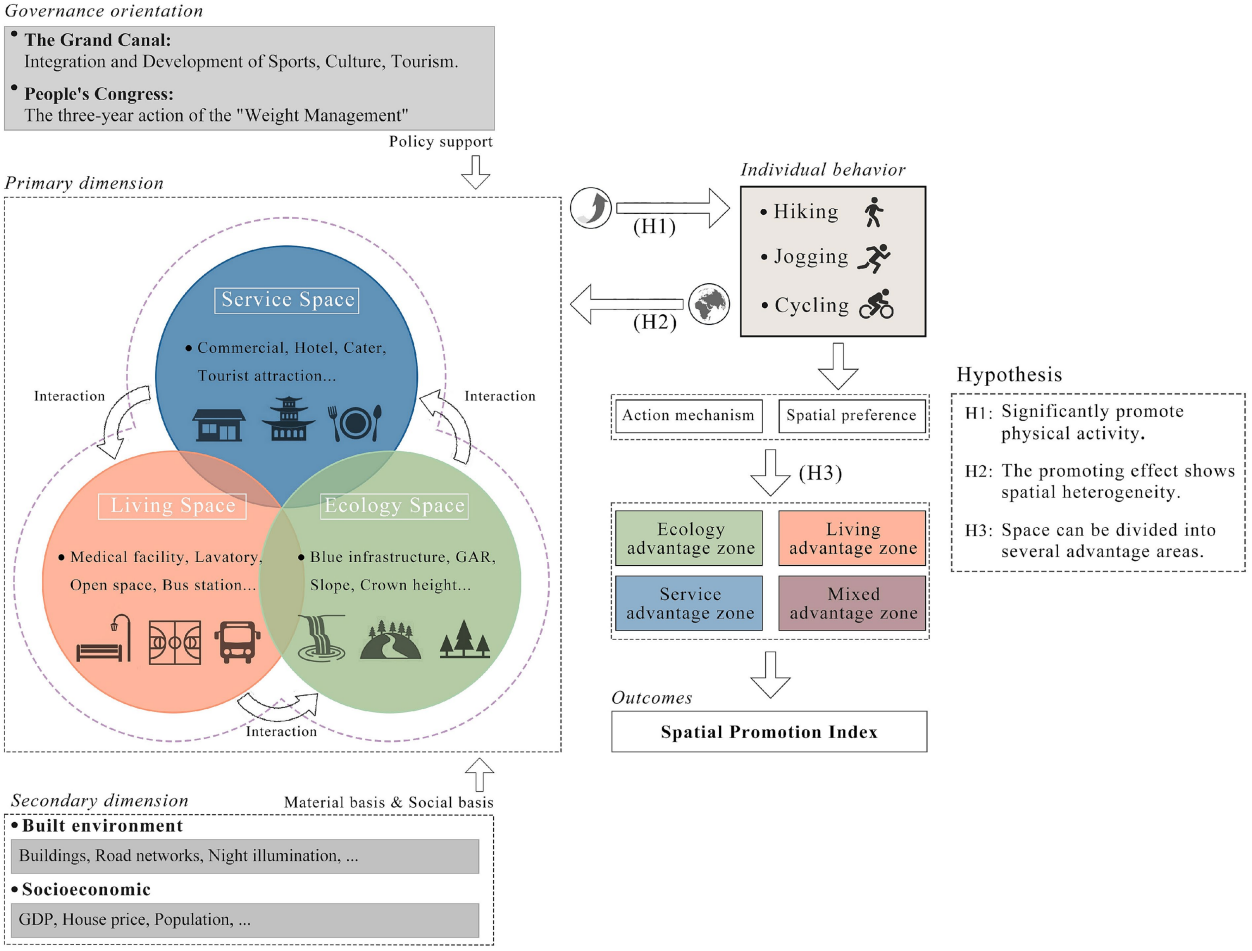
Theoretical framework and hypothesis.

The socio-ecological framework examines mechanisms that drive human behavior across multiple dimensions, government policy, physical environment, social culture, socioeconomic, and individual preferences—providing a multi-layered basis for understanding behavioral pathways (80). Relevant studies indicate that government policy and incentives can expand opportunities for public participation; physical environment directly shapes the cost and choice of activities; social culture and economic conditions modulate residents’ willingness and frequency of participation; and individual preferences determine sensitivity to environmental factors and selection tendencies (59, 81). Empirical work further supports these links, Cusack (82) reports significant effects of personal, social, and built-environment dimensions on active commuting, and Dai et al. (83) finds that policy, physical, and social environments strongly explain both participation rates and activity intensity. This study draws on socio-ecological systems theory to integrate multidimensional factors—including policy, environmental, and socioeconomic factors—within a unified analytical framework, with a particular focus on examining the combined effects of spatial-level factors on physical activity. Building on this framework (Figure 3), we propose the following hypotheses:

*H1*: Spatial factors exert significant promotion effects on physical activity and display threshold and interactive influences.

*H2*: The promotion effects of spatial factors on physical activity exhibit spatial heterogeneity, with different activity types showing distinct spatial preferences.

*H3*: Canal spaces can be partitioned into distinct advantage zones, permitting quantitative assessment of each zone’s capacity to promote physical activity and its spatial conversion efficiency.

#### 3.3.2 Data and factors

Spatial and trajectory data were compiled from multiple sources to construct the factor database. Specifically, we acquired spatial layers from the Geospatial Data Cloud and OpenStreetMap, and obtained trajectory records via the Keep API. The resulting variable system comprises one dependent variable, physical activity intensity, and five groups of independent variables: service, living, ecology, built environment, and socioeconomic. These five groups are further organized into three primary dimensions and two secondary dimensions according to empirical considerations and data availability.

Within the independent-variable set (Table 2), the service dimension includes tertiary-industry elements such as commercial services and cultural tourism. Street-front shops (31, 84) and commercial land parcels (30) have been shown to enhance joggers’ motivation and satisfaction. In addition, facility functional diversity, cultural appeal (85), and neighborhood character influence residents’ willingness to participate and activity intensity (29). The living dimension addresses the configuration of everyday facilities: the number of jogging trails (37), sports venues, and outdoor fitness parks are associated with higher levels of jogging and cycling, although greater quantity is not necessarily better (86). Some work suggests that excessive concentration of public facilities or highly developed public transport can paradoxically reduce residents’ willingness to be active (87). The ecological dimension focuses on natural resources, including green-space ratio (62), NDVI and canopy height (88). For higher intensity outdoor activities such as jogging and cycling, terrain undulation (89), access to country parks (55, 90), and blue-space accessibility (66, 91) are important. Within the built-environment and socioeconomic groups, variables such as road-network density, intersection density, and nighttime illumination intensity have been shown to relate closely to travel and activity patterns. Population density, housing prices, and GDP also tend to correlate positively with the intensity of activity clusters (60, 74). The indicators selected in this study primarily characterize canal-adjacent spaces in terms of functional layout, facility support, environmental exposure, and socioeconomic development. By emphasizing the relative differences among spatial units across multiple dimensions, they enable the identification of spatial associations between promoting factors and physical activity at the regional scale.

**Table 2:** Classification and quantification methods for independent variable factors.

Category	Abbreviation	Factors	Description or calculation
Service space	LUM	Land use mix	*H*′ = −Σ (Pi * log2 (Pi))
	CD	Commercial diversity	Same as above
	HAC	Hotel accommodation centrality	$\hat{f}(x)=(1/\mathrm{nh})\sum_{i=1}^{n}K((x-x_{i})/h)$
	CFC	Commercial facility centrality	Same as above
	CC	Catering centrality	Same as above
	TAC	Tourist attraction centrality	Same as above
	OCC	Office complex centrality	Same as above
	HSC	Historic streets centrality	Same as above
	NAS	Number of anchor store	Number of anchor store within the unit
Living space	EIC	Educational institution centrality	$\hat{f}(x)=(1/\mathrm{nh})\sum_{i=1}^{n}K((x-x_{i})/h)$
	MFC	Medical facility centrality	Same as above
	LC	Lavatory centrality	Same as above
	OSC	Open space centrality	Same as above
	DBS	Distance to bus station	Service area analysis from network analyst
	DSS	Distance to subway station	Service area analysis from network analyst
	NPL	Number of parking lots	Number of parking lots within the unit
	NT	Number of tracks	Number of tracks within the unit
	AFF	Accessibility of fitness facilities	Network analysis based on POI and road net
Ecology space	WBD	Water body density	Density = (water area/land area) * 100%
	LR	Length of riverbank	Length = Length of river center line * 2
	ABS	Accessibility of blue space	Network analysis based on POI and road net
	AGS	Accessibility of green space	Same as above
	GAR	Green area ratio	GAR = (green area/land area) * 100%
	NDVI	Normalized difference vegetation index	NDVI = (NIR − R)/(NIR + R)
	SG	Slope gradient	SG = (DEM/horizontal distance) * 100%
	AQ	Air quality	12-month mean PM 2.5 concentration
	CH	Crown height	Mean raster value of tree crown height
Built environment	BD	Building density	Density = (gross building area/land area) * 100%
	RND	Road network density	Density = (road length/land area) * 100%
	ID	Intersection density	Density = (intersection point/land area) * 100%
	II	Illumination intensity	Mean raster value of illumination intensity
Socio-economic	PD	Population density	Density = (population/land area) * 100%
	GDP	GDP density	GDP density = total regional GDP/land area
	HP	House price	Mean second-hand house price

The dependent variables comprised three categories of physical activity—hiking, jogging, and cycling (Table 3). Activity trajectories were sourced from Keep, which had over 29 million monthly active users at the time of data collection. Weekend user trajectory data were collected over a total of 24 days between April and June 2024. The sample therefore captures the behavioral characteristics of active Keep users during the spring season. In the Beijing–Tianjin–Hebei region, spring is characterized by rising temperatures and relatively favorable outdoor conditions, making it a peak period for outdoor physical activities such as walking, jogging, and cycling. Compared with the high temperatures of summer and the low temperatures and strong winds of autumn and winter, spring weekends provide more stable conditions for outdoor exercise and are thus suitable for collecting trajectory data on physical activity. In addition, Keep users generally show a stronger propensity for proactive exercise and higher expectations regarding environmental quality than the general population. Therefore, this dataset is particularly well suited to examining moderate- to high-intensity linear physical activities and to exploring spatial association patterns between corridor environments and physical activity.

**Table 3:** Classification and quantification methods of dependent variable factors.

Category	Abbreviation	Factors	Description or calculation
Physical activity	H	Hiking intensity	Outdoor hiking and walking activities
	J	Jogging intensity	Outdoor jogging without the aid of tools
	C	Cycling intensity	Outdoor cycling events not for commuting

After obtaining the trajectory data, this study first preprocessed the raw records. Records with durations of less than 5 min were excluded to reduce interference from non-genuine exercise entries, such as test uploads. Pace anomalies were then filtered using reasonable speed ranges for different activity types, and trajectories with abnormal point jumps or positioning discontinuities were removed. After this cleaning process, 29,400 valid trajectories remained. We use cumulative activity time per unit area to characterize the level of physical activity within each spatial unit. By integrating activity frequency with travel distance and speed, it estimates the cumulative activity time of hiking, jogging, and cycling across different spatial units, and then standardizes these values by unit area to reflect the carrying capacity and concentration of physical activity in different areas. The calculation formula is as follows:


$$I_{A}=\frac{N.d}{v.A}$$ (1)

In the formula, $I_{A}$ denotes physical-activity intensity; *N* is the number of activity occurrences; *d* is the total distance covered; *v* is the average speed, calculated from the distance and duration recorded in Keep’s activity logs; and *A* is the unit area.

#### 3.3.3 Data preprocessing and impact factor system construction

**Figure 4:**
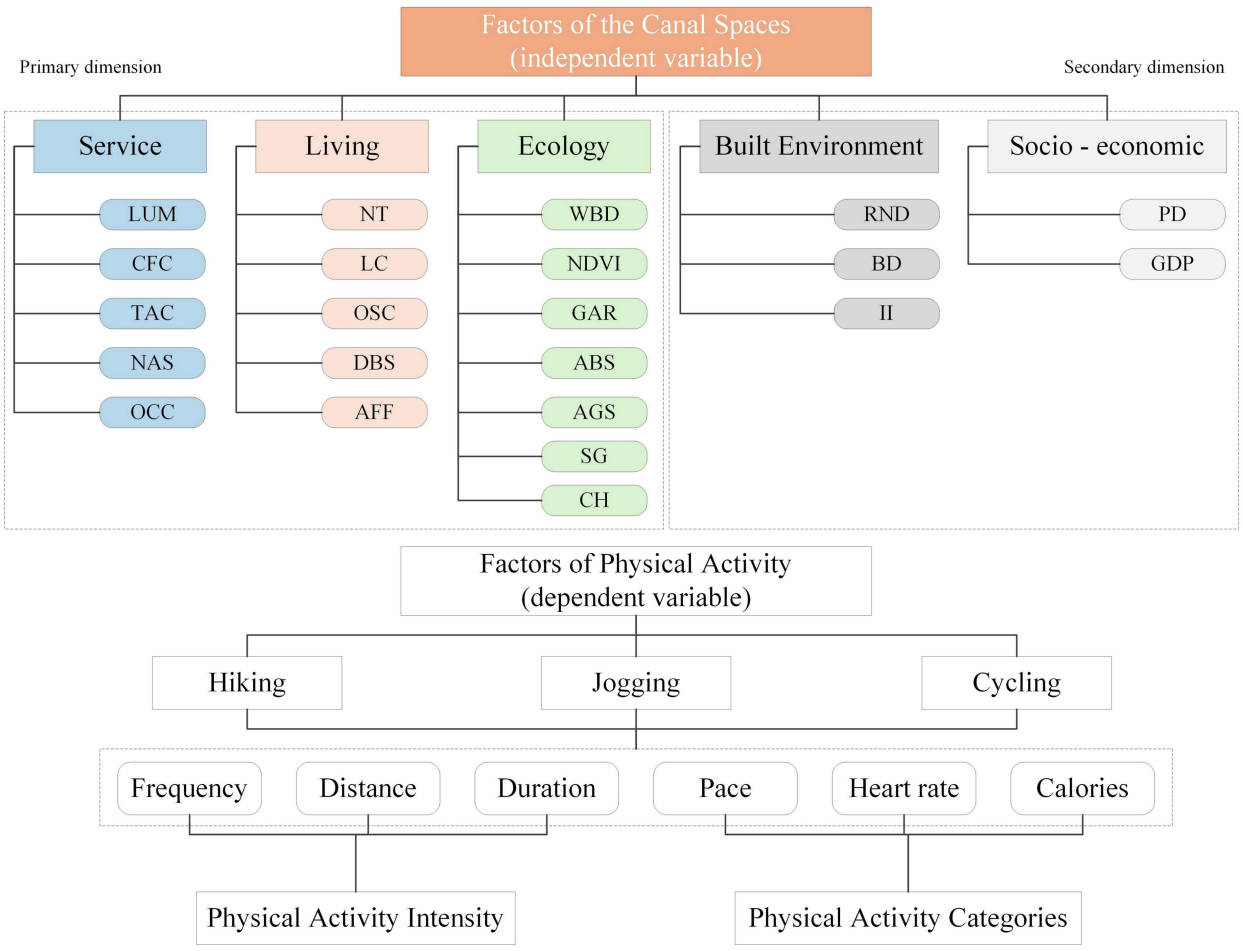
Physical activity impact factors system along the canal-adjacent space.

To remove multicollinearity, we first tested latent-factor correlations using Pearson correlation analysis and applied principal component analysis (PCA) for dimensionality reduction. Factors were retained according to explained variance ratio and principal component loadings to ensure independence within each dimension. After validation with Pearson correlation analysis, variance contribution rates were used to derive indicator weights for the subsequent weighted spatial clustering. Based on the principal component analysis results, 12 highly correlated indicators were excluded. The final spatial factors system for physical activity along the waterway therefore comprised five service factors, five living factors, seven ecology factors, three built environment factors, and two socioeconomic factors (Figure 4).

### 3.4 Methods for promoting factor identification and promotion index quantification

First, we employ an XGBoost regression model to identify the key factors that promote physical activity in the canal-adjacent space and to explore their nonlinear relationships. We then use Shapley values to quantify each factor’s contribution to model outputs. An information-gain procedure is subsequently applied to convert Shapley contributions into factor weights, thereby quantifying each promoting factor’s contribution within the multidimensional space. The calculation formula is given below:


$$\mathrm{Va}r_{\text{total}}=\frac{1}{n}\sum_{i=1}^{n}{\left(y_{i}-\bar{y}\right)}^{2}$$ (2)

$$\mathrm{Gai}n_{f}=\begin{array}{c} \max\\ t \end{array}[\mathrm{Va}r_{\text{total}}-(\frac{|L|}{n}\mathrm{Var}(\mathrm{yL})+\frac{|R|}{n}\mathrm{Var}(\mathrm{yR}))]$$ (3)

$$\omega_{i}=\frac{\mathrm{Gai}n_{\mathrm{fi}}}{\sum_{j=1}^{k}\mathrm{Gai}n_{j}}$$ (4)

In the formula, ${\mathrm{Var}}_{\text{total}}$ denotes the total variance of the target variable, and ${\text{Gain}}_{f}$ denotes the gain of feature *f*. *L* is the set of samples with Shapley values below the threshold, and *R* is the set of samples with Shapley values above the threshold. $\omega_{i}$ denotes the Shapley weight for sample.

Second, multiscale geographically weighted regression (MGWR) and K-means clustering were employed to identify the spatial heterogeneity of promoting factors and the dominant spatial categories. MGWR was conducted at the county level to capture the regional-scale spatial non-stationarity of the promoting factors, whereas spatial clustering was performed using 2.5 × 2.5 km grid cells to characterize local functional combinations and dominant activity patches along the canal corridor. On this basis, canal-adjacent spaces were classified into four functional types: service-dominated, living-dominated, ecology-dominated, and mixed-function spaces. The classification formula is provided below:


$$S_{t}=\frac{1}{n}\sum_{i=1}^{t}C_{i}$$ (5)

$$\text{Spac}e_{\text{Type}}=\{\begin{array}{cc} \mathrm{Mix}\;\text{Space}, & \text{if}\;S_{\max}-S_{\sec\mathrm{ond}}<\theta .S_{\max}\\ \text{Type corresponding to}\;S_{\max}, & \text{otherwise} \end{array}$$ (6)

In the formula, $S_{t}$ denotes the spatial score for type *t*; $S_{\max}$ is the highest spatial score, $S_{\text{second}}$ the second-highest, and θ the threshold ratio. A spatial unit is classified as a mixed space when the difference between the highest and second-highest scores is less than 20% of the highest score.

Finally, the Natural Breaks method is used to partition the study area into physical activity advantage and disadvantage zones. Integrating the promoting factor weights derived above, we compute a physical activity promotion index for the canal corridor using a weighted-average approach. The calculation formula is presented below:


$$I_{i}=\frac{P\sum_{j=1}^{n}S_{\mathrm{ij}}\omega_{\mathrm{ij}}}{\sum_{i\prime =1}^{m}[P\sum_{j=1}^{n}S_{\mathrm{ij}}\omega_{\mathrm{ij}}]}$$ (7)

In the formula, $I_{i}$ denotes the spatial promotion index for spatial type *i*; *j* indexes the corresponding factors. $S_{\mathrm{ij}}$ is the sample’s score for the jth factor in spatial type *i*; and $\omega_{\mathrm{ij}}$ is the Shapley-derived weight for that factor; *P* denotes the physical activity intensity score for the sample.

## 4 Results

### 4.1 Impact factor analysis

#### 4.1.1 Factor importance

This study employed an XGBoost regression model to comprehensively model the impact factors. Results indicated that moderating variables, particularly population density and illumination intensity, exerted consistent effects across all three physical activity types. High-intensity activities (jogging and cycling) showed stronger sensitivity to favorable nighttime lighting. Distinct response patterns also emerged by activity: hiking was mainly influenced by factors within the living and ecology dimensions; jogging exhibited demand across service, living, and ecology dimensions; and cycling was driven predominantly by ecology dimension factors.

**Figure 5:**
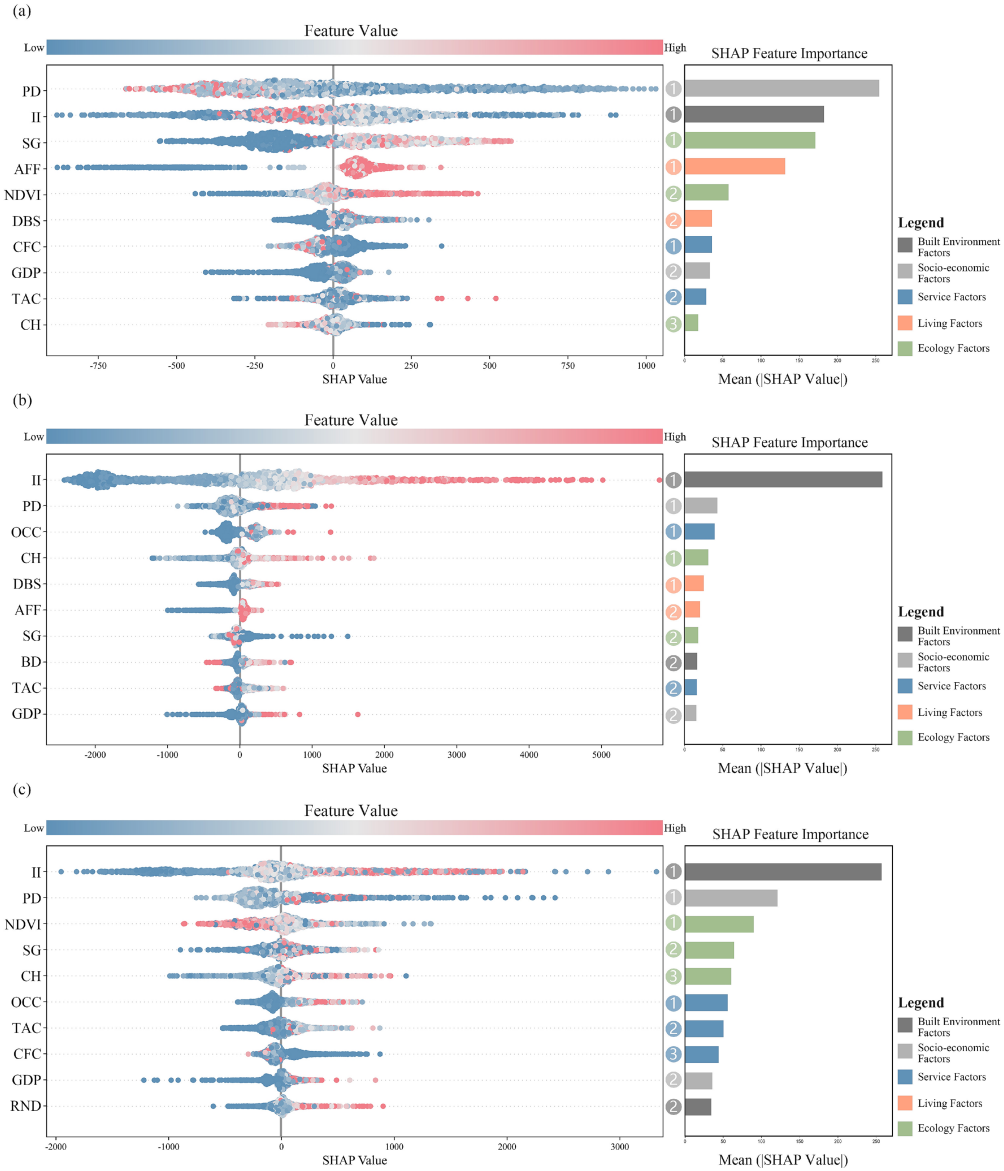
Ranking of the importance of impact factors: **(a)** The importance of impact factors on hiking; **(b)** The importance of impact factors on jogging; **(c)** The importance of impact factors on cycling.

Hiking preferences were broadly categorized into two types (Figure 5a): wilderness hiking, which favors natural environments, and urban-leisure hiking, which favors urban settings. However, in most samples hiking was concentrated outside urban cores and showed negative correlations with population density and illumination intensity. The positive associations with slope and NDVI further indicate that hikers prefer natural or semi-natural spaces away from cities (51). Jogging was mainly promoted by nighttime illumination and was concentrated within urban built-up areas (Figure 5b). As a low-barrier, route-flexible activity, jogging more readily attracted white-collar populations and tended to occur on urban roads with high canopy cover and gentle topography. Furthermore, GDP is positively correlated with jogging intensity, suggesting that economically developed areas tend to provide better public spaces, infrastructure, and environmental amenities, thereby offering more favorable conditions for jogging (92). Cycling was driven primarily by ecology and service factors (Figure 5c), showing positive correlations with illumination intensity, slope gradient, and canopy height, although negative correlations appeared in some samples, likely reflecting differences in cycling modes. High night illumination and low vegetation cover improved visibility and perceived safety during higher-speed movement, while slope met training-load requirements.

#### 4.1.2 The effect threshold of physical activity promoting factors along the canal space

Nighttime illumination intensity was a key environmental driver of physical activity, showing distinct threshold effects by activity type: it tended to promote high intensity activities while inhibiting nature-oriented pursuits.

**Figure 6:**
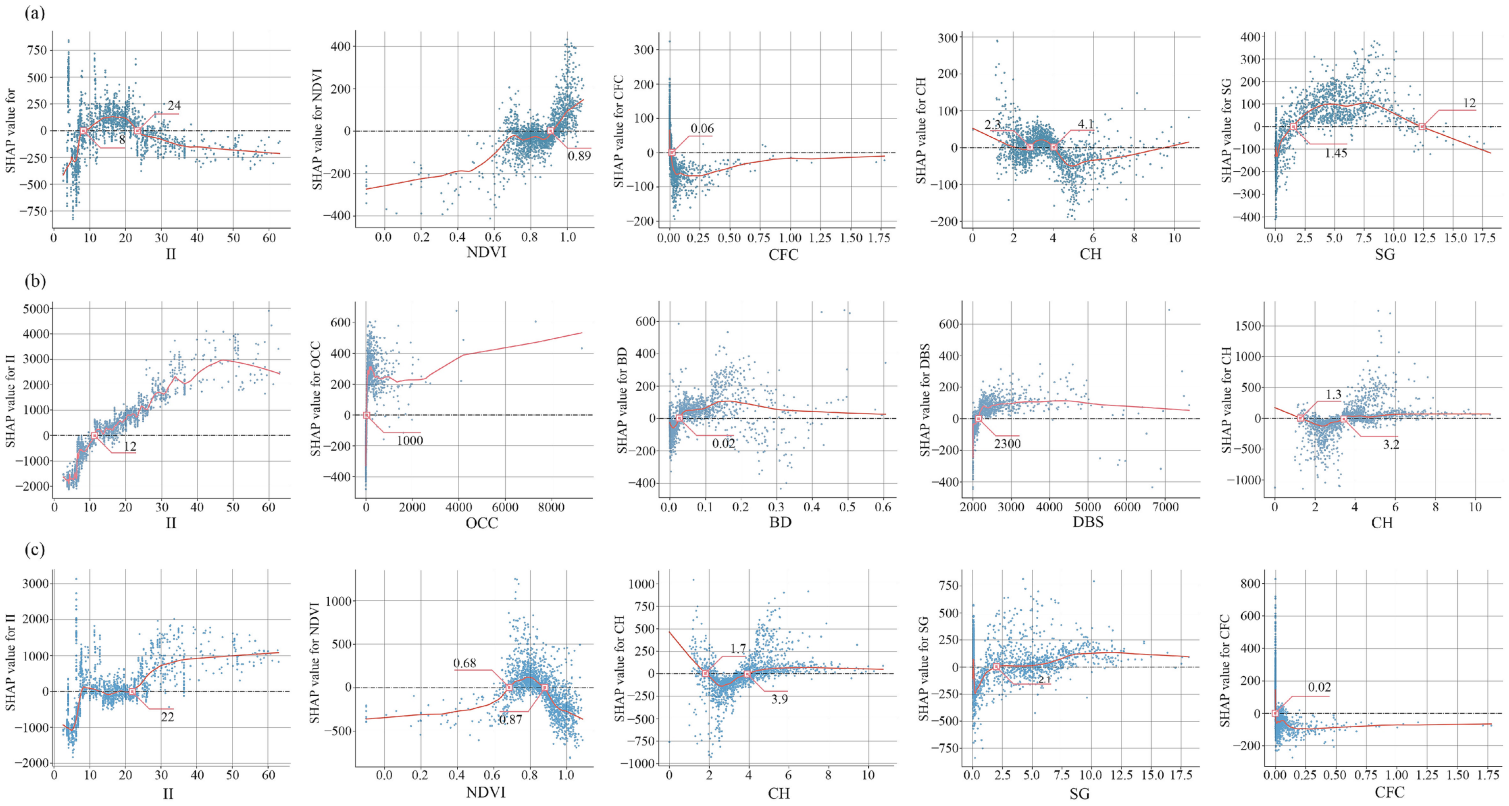
Threshold effects of key promoting factors on physical activity: **(a)** Threshold of key promoting factors on hiking; **(b)** Threshold of key promoting factors on jogging; **(c)** Threshold of key promoting factors on cycling.

For hiking, illumination intensity followed a dual-threshold response (Figure 6a): when intensity fell below about 8 lux the effect was unstable, whereas intensities above about 24 lux were associated with inhibitory effects. Combined thresholds for NDVI and commercial facility centrality indicated that hiking mainly occurred in densely vegetated areas and depended more on ecological conditions than on commercial services, promotion observed when NDVI > 0.89 and canopy height about 2.3–4.1 m. Slopes between 1.45° and 12° met the hiking-demand threshold. Jogging favored well-lit environments and was sensitive to threshold effects of office complex centrality, building density, distance to bus station, and canopy height (Figure 6b). As a form of daily exercise, jogging places high demands on environmental safety (58). Areas with a high concentration of office buildings typically provide well-developed road networks, adequate lighting, and supporting infrastructure, and therefore show a strong association with jogging (Figure 6c). For example, when nighttime illuminance is below 22 lux, its effect on jogging remains unstable; once this threshold is exceeded, the promoting effect becomes markedly stronger and rapidly approaches saturation. Unlike hiking, cycling exhibited strong nonlinear responses to vegetation cover and canopy height: overly dense vegetation can obstruct visibility and impair safe decision-making at higher speeds, whereas ground slopes above about 2.1° positively promoted cycling by meeting training-load needs. Cyclists also tended to avoid areas with very intense nighttime illumination intensity and high commercial density, consistent with established path-selection findings (93).

#### 4.1.3 Interaction effects of physical activity promoting factors along the canal space

Illumination intensity exhibits a significant nonlinear relationship with physical activity: the direction and magnitude of its effect vary by activity type and by environmental context. Furthermore, illumination interacts with factors such as NDVI, slope gradient, canopy height, and population density to produce synergistic promotion, antagonistic inhibition, and regulatory-amplification effects on activity levels.

**Figure 7:**
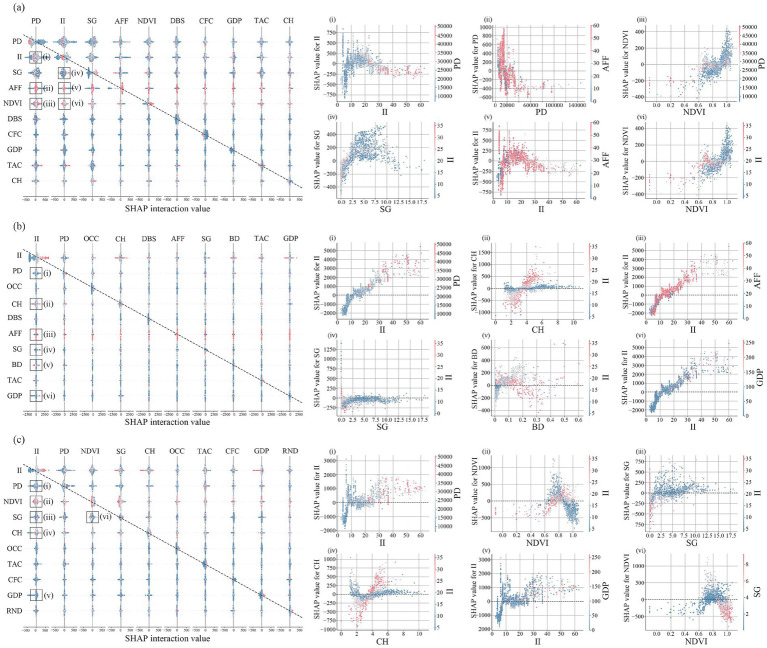
The interaction effect of key promoting factors on physical activity: **(a)** Hiking interaction matrix and SHAP dependence plots; **(b)** jogging interaction matrix and SHAP dependence plots; **(c)** Cycling interaction matrix and SHAP dependence plots.

During hiking (Figure 7a), nighttime illumination intensity and population density both exerted inhibitory effects on hiking and showed a synergistic negative interaction. Illumination intensity also interacted antagonistically with NDVI and slope gradient: higher vegetation cover and steeper slopes significantly mitigated the inhibitory effect of illumination. Moreover, even in areas with generally high fitness facilities accessibility, population density remained negatively associated with hiking intensity, indicating that fitness facilities were not the primary destinations for hikers in high-density built environments. For jogging (Figure 7b), Illumination intensity markedly moderated interaction effects: it amplified its inhibitory role when paired with negative factors and strengthened its promotional role when paired with positive factors. For example, canopy height had little influence under low illumination but, under high illumination, canopy height substantially amplified jogging activity; the direction of canopy height’s effect did not depend on illumination level. Similar modulation patterns were observed for slope gradient and building density effects. For cycling (Figure 7c), interactions with illumination illumination were more complex. As NDVI increased from low to high, the effect of illumination on cycling followed a nonlinear pattern: the interaction was strongest in sparsely vegetated areas; in moderately vegetated zones, ecological comfort dominated and weakened illumination’s marginal impact; in areas of very dense vegetation, where visibility was obstructed, NDVI itself inhibited cycling, reducing the interactive contribution of illumination. Cyclists also tended to select routes with moderate slopes and low lighting impact, avoiding heavily vegetated or steep natural environments.

### 4.2 Spatial heterogeneity of promoting factors

**Figure 8:**
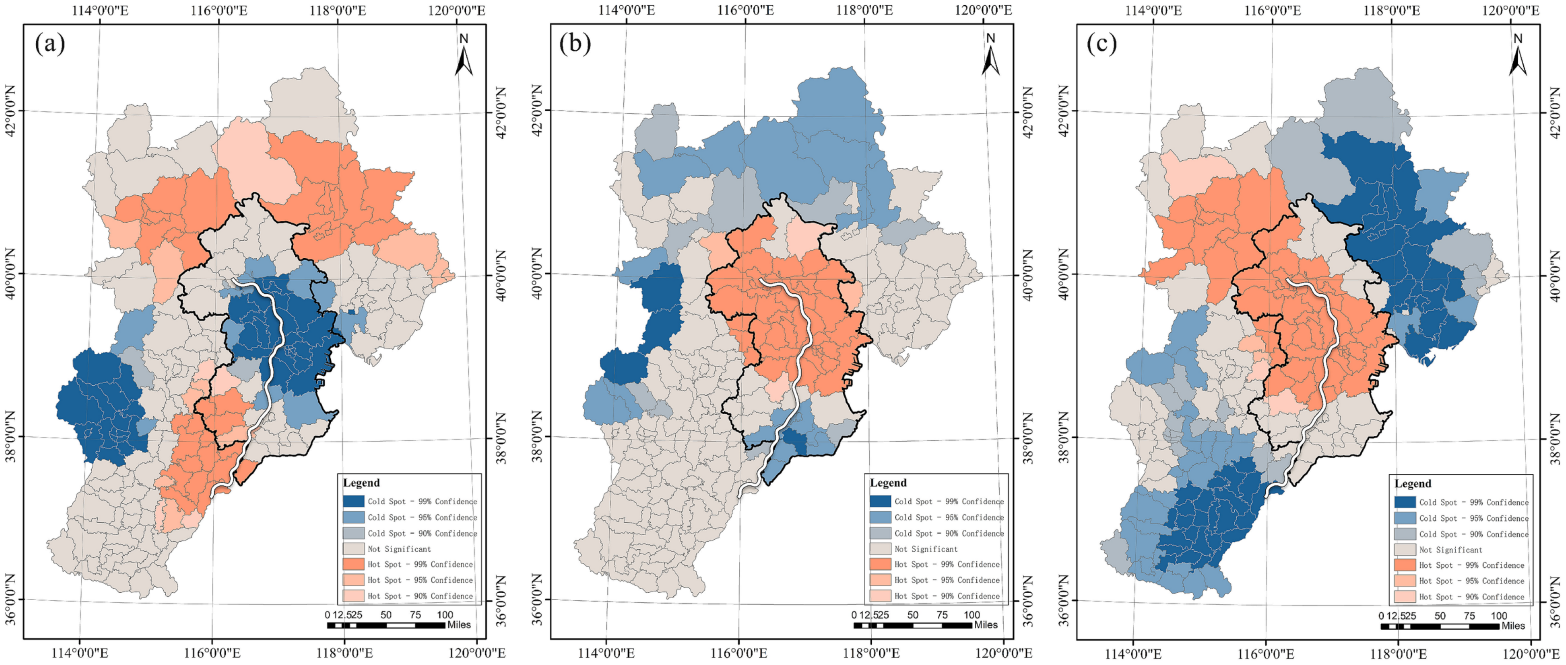
Spatial aggregation characteristics of physical activity: **(a)** Spatial aggregation of hiking; **(b)** Spatial aggregation of jogging; **(c)** Spatial aggregation of cycling.

The Beijing–Tianjin–Hebei section of the Grand Canal spans 58 county-level administrative units, which show substantial regional variation in population density, economic development, infrastructure provision, and policy context (94, 95). Accordingly, this study adopts county-level administrative units as the basic analytical scale for identifying the regional spatial differentiation of promoting factors. To test the spatial dependence of physical activity, we conducted spatial autocorrelation analyses for hiking, jogging, and cycling. The results show that all three activity types exhibit significant spatial clustering (Table 4), while hotspot analysis further reveals multi-scale differences in clustering patterns among the three activities (Figure 8). Accordingly, we applied MGWR to examine the multi-scale spatial heterogeneity of activity responses to the impact factors, and used local T-tests to identify statistically significant and practically meaningful influence zones.

**Table 4:** Spatial autocorrelation test.

Physical activity	Moran’s *I*	*p*	*Z*-score
Hiking	0.585	0.000	20.935
Jogging	0.813	0.000	30.001
Cycling	0.759	0.001	27.082

**Figure 9:**
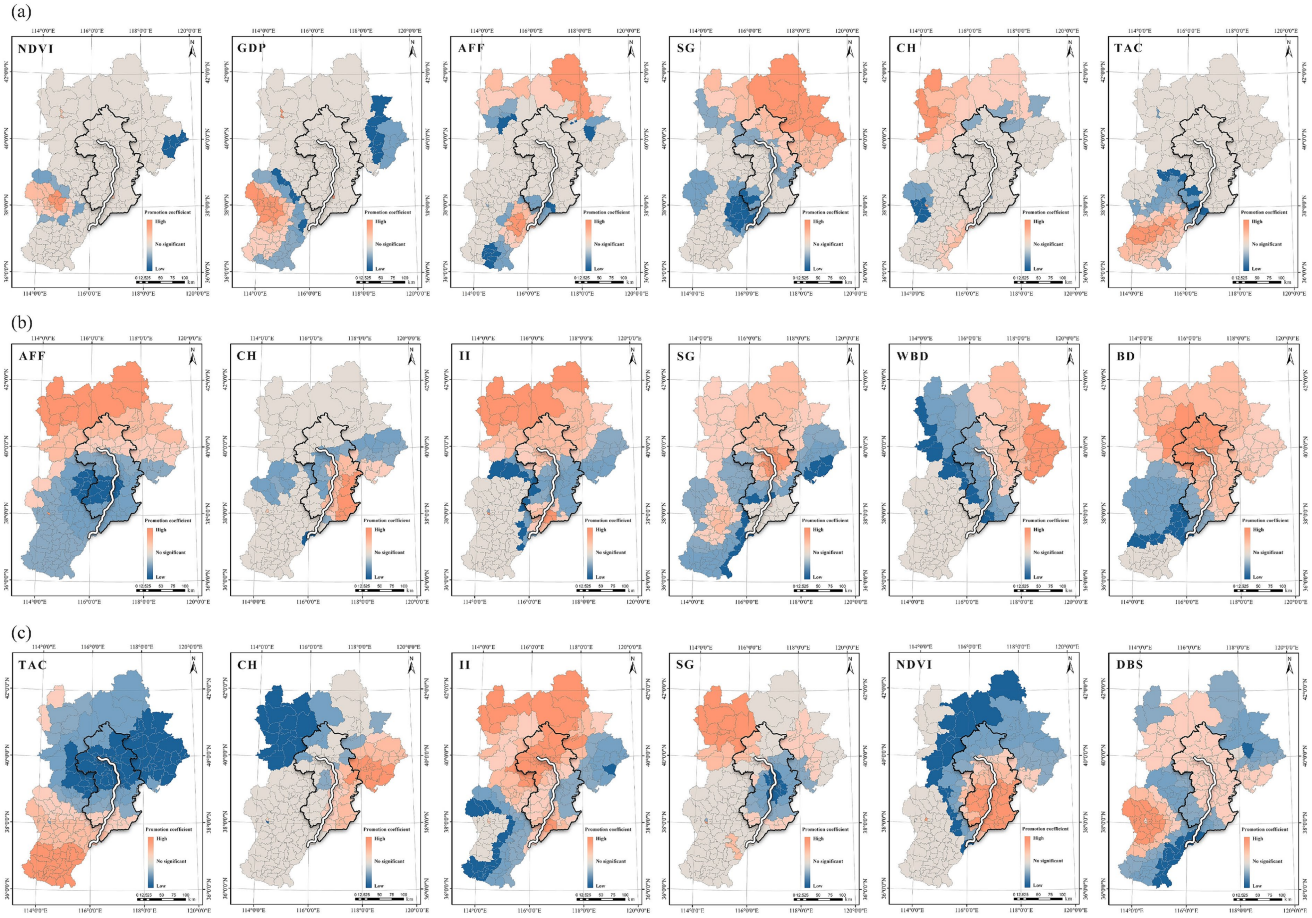
Spatial heterogeneity of promoting factors: **(a)** Promotion factors for spatial heteroge-neity in hiking; **(b)** Promotion factors for spatial heterogeneity in jogging; **(c)** Promotion factors for spatial heterogeneity in cycling.

During hiking (Figure 9a), the promoting effect between NDVI and GDP per capita showed a multi-scale distribution, concentrated mainly in the southwestern urban belt of the Beijing–Tianjin–Hebei (e.g., Shijiazhuang, Xingtai, and Handan). Slope gradient effects were stronger in the north and weaker in the south, gradually diminishing across the lower-reaches plain areas along the Grand Canal, reflecting a preference for hiking in areas with prominent natural landscapes and varied topography. For jogging (Figure 9b), fitness facilities accessibility, canopy height, and illumination intensity produced significant positive impacts across most counties, whereas slope, water body density, and building density generally had inhibitory effects. The promotional effect of fitness facilities declined from northwest toward the northeast–central zones, and the enhancing role of lighting was especially pronounced in northern, ecologically dense areas. Zones where canopy height promoted jogging closely overlapped with canal-side and coastal spaces, indicating that shaded, waterfront conditions supported higher rates of waterfront jogging. For cycling (Figure 9c), canopy height significantly promoted cycling in eastern coastal areas, while the influence of illumination intensity was stronger in northern parts of the region and along canal corridors. NDVI effects showed spatial differentiation: within the relatively homogeneous-vegetation core of the canal corridor, vegetation’s role shifted toward inhibition—consistent with cyclists’ sensitivity to visual permeability and path-safety concerns. This pattern aligns with prior findings on path-selection mechanisms (96).

### 4.3 Zoning of physical activity advantage zones

**Figure 10:**
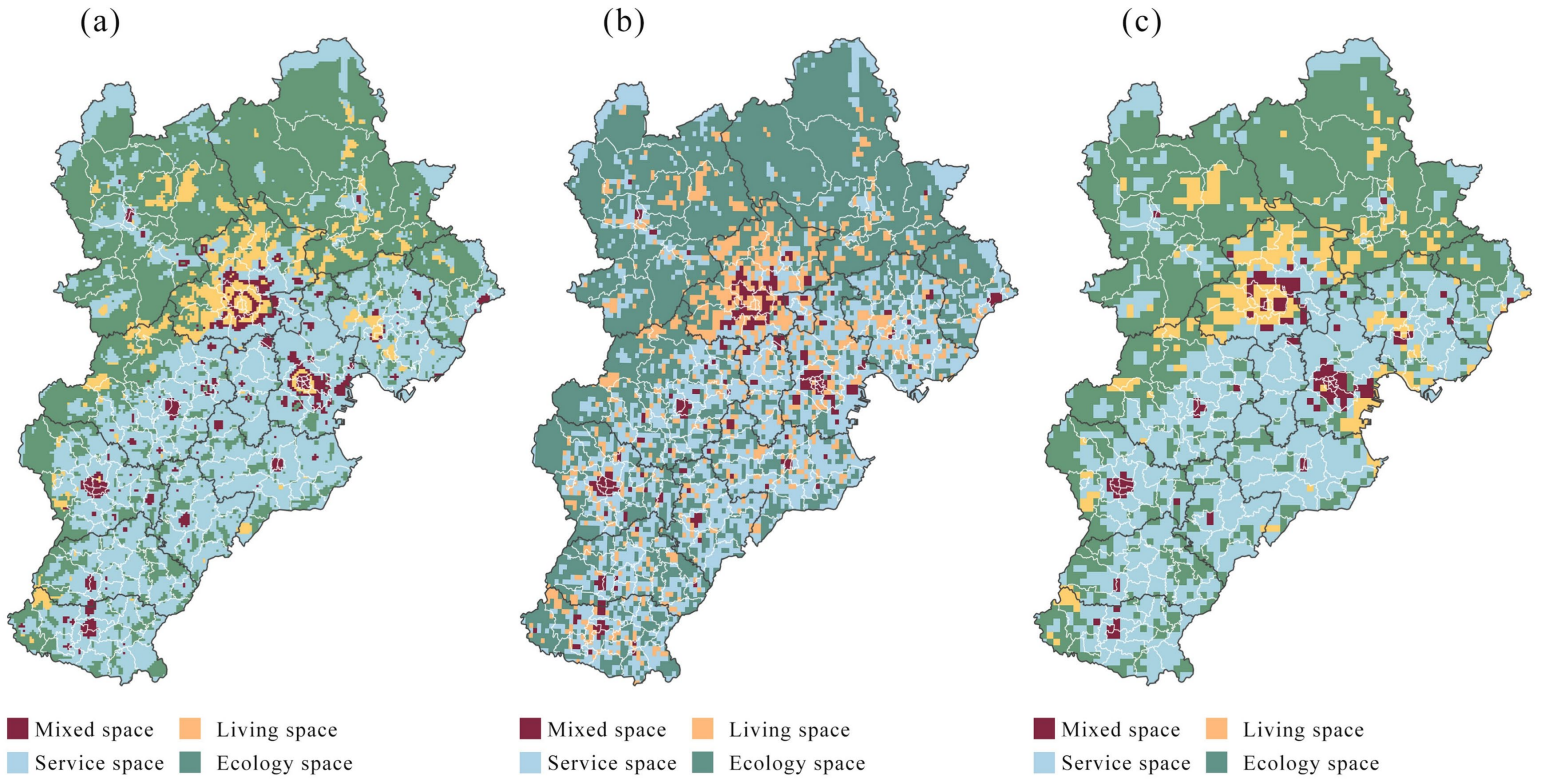
Spatial unit scale sensitivity analysis: **(a)** 1 × 1 km scale space grid; **(b)** 2.5 × 2.5 km scale space grid; **(c)** 5 × 5 km scale space grid.

Building on the county-level identification of spatial heterogeneity, this study further adopts a grid-based approach to characterize local functional combinations and the distribution of physical-activity advantage zones along the canal corridor, thereby revealing the distinctive spatial characteristics of different advantage-zone types at the unit level. To examine the scale sensitivity of the spatial clustering results, we compared three spatial resolutions: 1 × 1 km, 2.5 × 2.5 km, and 5 × 5 km (Figure 10). As the spatial scale changed, the clustered patches showed some variation in morphology, number, and extent, indicating a certain degree of scale sensitivity. However, from an overall perspective, the distribution patterns of spatial entities, the locations of core agglomeration areas, and the broader regional structure remained largely consistent across scales, suggesting that the clustering results are robust under different spatial resolutions.

#### 4.3.1 Identification of physical activity advantage zones

**Figure 11:**
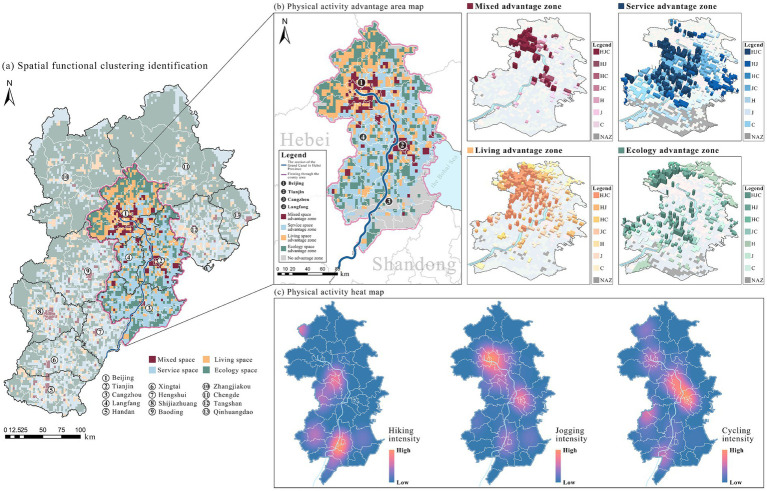
Spatial clustering and partitioning: identifying advantage zones for physical activities along the Grand Canal: **(a)** Spatial functional clustering identification; **(b)** Physical activity ad-vantage zone; **(c)** Physical activity heat map.

Using K-Means clustering to classify 9,503 spatial units (Figure 11a), the results showed the following patterns along the Grand Canal corridor within the Beijing–Tianjin–Hebei. Ecology spaces accounted for the largest share (52.9%) and displayed a northwest-concentrated, corridor-penetration pattern: 72% of these units lay in the Taihang Mountain zone (including Chengde and Zhangjiakou), while the remaining 28% occurred along the Beijing–Tianjin green corridor and urban–rural transition areas. Service spaces represented 29.5% and were scattered across the southeast, forming a cluster belt running from Langfang through Tianjin to Cangzhou along the middle and lower reaches. Living spaces comprised 11.7%, mainly embedded within urban built-up areas, while mixed spaces concentrated around major urban centers such as Shijiazhuang, Beijing, and Tianjin.

Overlaying spatial-type clusters with physical-activity distributions identified physical activity advantage zones (Figure 11b). Multi-activity promotion zones clustered in the canal’s core: mixed-function and living-dominated zones concentrated in the metropolitan core, whereas service- and ecology-dominated multi-activity zones were distributed along the middle and lower reaches. In general, both the diversity of supported activity types and activity intensity declined with distance from the canal, diminishing from core through transition to peripheral zones (Figure 11c).

#### 4.3.2 Identification of promoting factors in advantage zones

**Figure 12:**
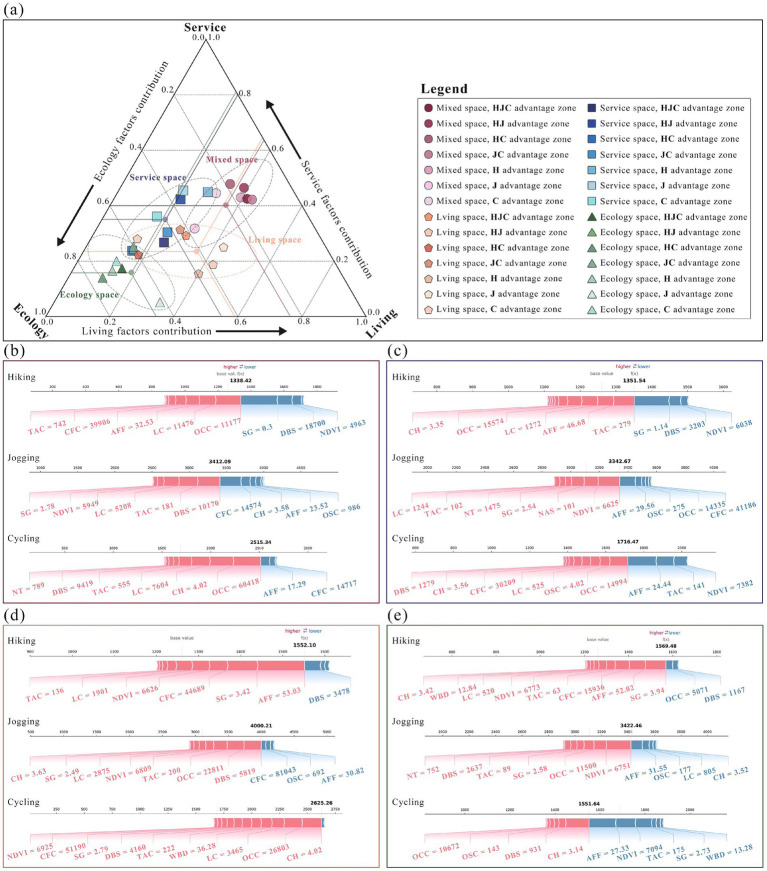
The three-dimensional composition of promoting factors in advantage zones: **(a)** Contribution of primary dimensions to advantage zones of physical activity; **(b)** Mixed ad-vantage zone; **(c)** Service advantage zone; **(d)** Living advantage zone; **(e)** Ecology advantage zone.

To examine how promoting factors differ by spatial context, we subdivided physical activity advantage zones into 28 categories for detailed analysis. Results show that key promoting factor s in mixed advantage zones primarily derive from the service and living dimensions (Figure 12a). In service advantage zones, dominant promoting factors include tourist attraction centrality, NDVI, and office complex centrality. Living advantage zones are mainly driven by canopy height, fitness facilities accessibility, and distance to bus stations. Ecology advantage zones are dominated by slope gradient, NDVI, and canopy height.

Promoting factors often exhibit cross-dimensional characteristics (Figure 12b). Taking the cycling advantage zone as an example, its promoting factors simultaneously encompass canopy height, office complex centrality, and lavatory centrality. This indicates that maximizing spatial efficiency relies on the synergistic interaction of multidimensional factors rather than local optimization within a single dimension. Vertical comparisons further reveal that the promoting effects of factors are modulated by their spatial context, reflecting users’ differentiated expectations for spatial functions across environments. For instance, in the hiking model, slope gradient and office complex centrality exhibit opposite effects in service advantage and ecology advantage zones (Figures 12c and 12e). Similarly, in the jogging model, restroom centrality and canopy height display mirrored effects between living advantage and ecology advantage zones (Figures 12d and 12e).

### 4.4 Quantification of physical activity promotion index in advantage zones

**Figure 13:**
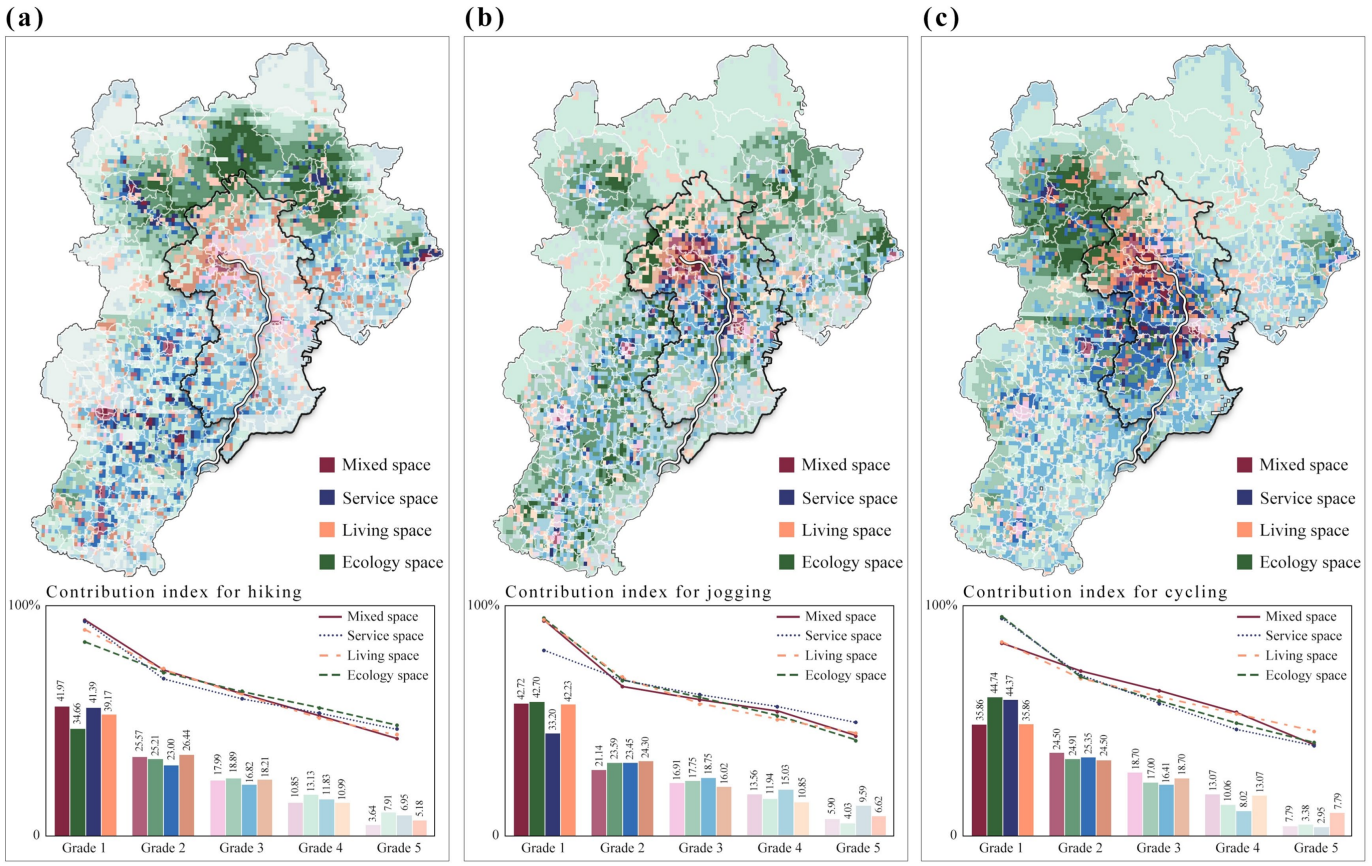
Spatial pomotion index distribution and intensity grading: **(a)** Spatial promotion index intensity for hiking; **(b)** Spatial promotion index intensity for jogging; **(c)** Spatial promotion in-dex intensity for cycling.

Based on the foregoing analyses, we computed physical activity promotion indices for the four spatial categories using a weighted average method (Figure 13). In high-promotion zones for hiking, ecology and living spaces formed concentrated, contiguous clusters, whereas service and mixed spaces were more dispersed across the southern portion of the Beijing–Tianjin–Hebei and the lower reaches of the Grand Canal. Notably, continuous ecology advantage zones in the north exhibited substantially higher hiking promotion scores than canal side and southern areas. High-promotion zones for jogging clustered mainly around urban centers along the canal, with mixed, service, and living spaces showing strong promotion levels, while ecology spaces displayed a more scattered pattern. High-promotion zones for cycling formed an axial belt extending from the ecology advantage zone in Zhangjiakou through Beijing and Tianjin to Cangzhou.

The distribution trend of the promotion index is illustrated in a line chart, with the slope representing the concentration of the physical activity promotion index. The curve for hiking ecology advantage zones shows a relatively gentle slope, indicating a more even distribution of the promotion index. In contrast, the curve for living and mixed advantage zones is steeper, suggesting that the promotion effect for hiking is more concentrated in higher-level areas. On the other hand, jogging and cycling follow the opposite pattern. This suggests that hiking, which emphasizes nature experiences, is more reliant on landscape quality and topographical conditions, leading to a dispersed and balanced distribution of its promotion effects across ecology zones. In contrast, jogging and cycling, which prioritize continuity, safety, and services, are more dependent on integrated road networks, lighting, and supporting facilities. As a result, their promotion effects are more concentrated in urban living and mixed areas.

**Figure 14:**
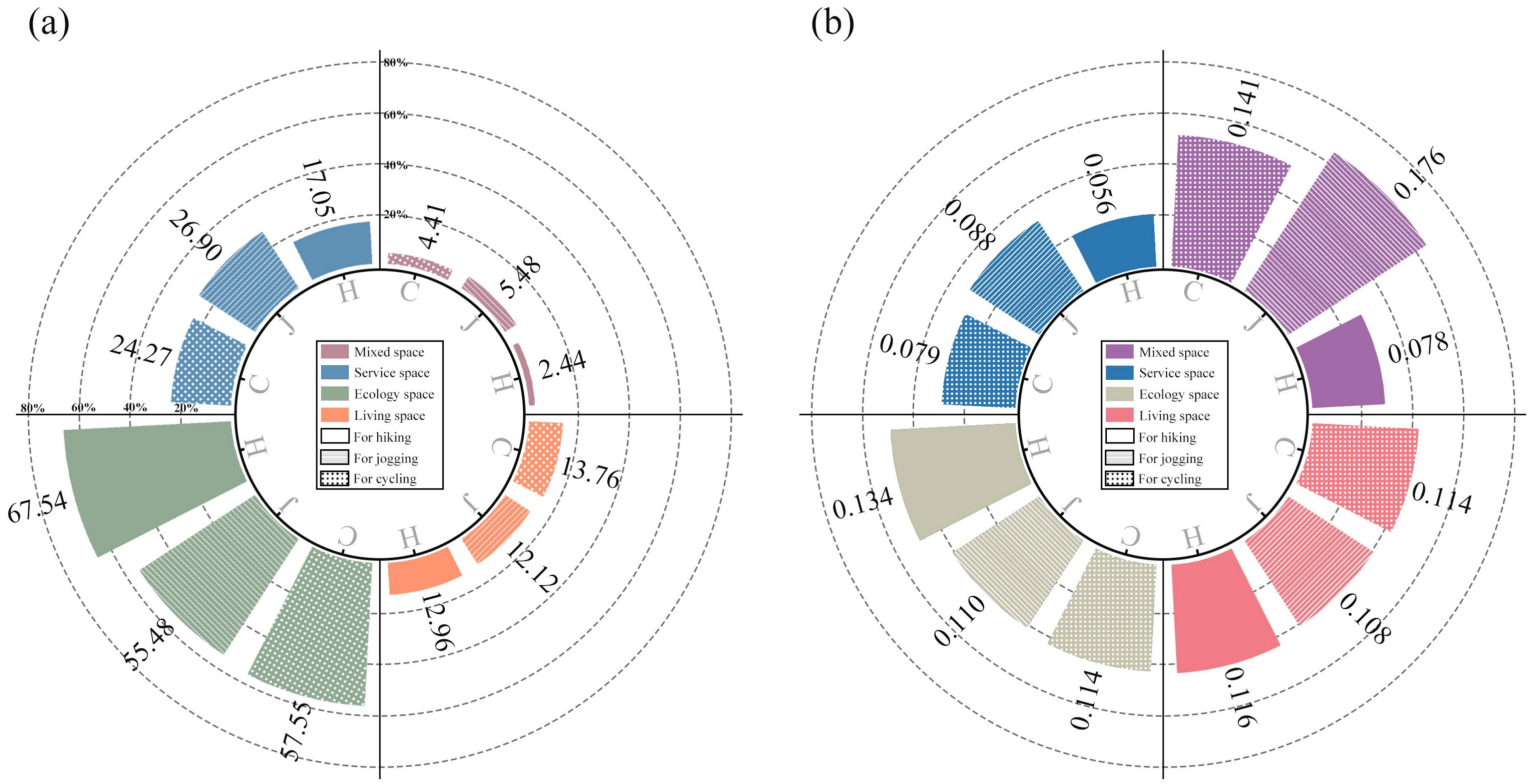
Spatial promotion index and spatial conversion efficiency distribution: **(a)** The overall promotion index to physical activity; **(b)** The spatial conversion efficiency to physical activity.

From a broader perspective (Figure 14), ecology spaces cover the largest area and exhibit the highest overall promotion index across all three physical activity categories. They account for approximately 67% of hiking promotion and more than 50% for both jogging and cycling promotion. In contrast, living and service spaces, primarily located in urban and suburban areas, show relatively minor overall promotion effects, contributing only about 12–26%. While mixed spaces have a smaller total area and a lesser overall promotional impact, their efficiency becomes apparent when calculating the spatial conversion efficiency, especially for jogging activities. This suggests that jogging benefits more from multifunctional, composite spaces than from single-function areas.

## 5 Discussion

### 5.1 Main findings interpretation

This study examines how spaces along the Beijing–Tianjin–Hebei section of the Grand Canal promote three types of physical activity: hiking, jogging, and cycling. The analysis covers 58 counties across 13 cities in three provincial-level regions. It investigates the key promoting factors and spatial distribution of physical activity intensity from the perspectives of services, daily living, ecology, built environment, and socioeconomic conditions.

The results indicate that hiking activities are primarily concentrated in suburban areas such as Zhangjiakou and Chengde, as well as ecological corridors downstream. Jogging is more concentrated in urban canal sections and is highly dependent on commercial, cultural, and residential facilities. Cycling tends to favor linear, continuous greenway networks, and is distributed across both urban and rural areas. Overall, the closer to cities, the richer the environmental elements along the canal, which support a wider range of physical activities, making these areas high-frequency zones for hiking, jogging, and cycling. Additionally, areas with higher incomes and greater population density generally provide better sports facilities, public spaces, and overall environmental quality, and accordingly show higher levels of physical activity. By contrast, low-income and sparsely populated areas tend to lack environmental safety and supporting resources, leading to relatively weaker spatial clustering of physical activity (97, 98).

Nighttime illumination intensity, building density, and road network density are the most significant promoting factors associated with physical activity along the canal corridor. Among these, jogging and cycling show stronger responses to nighttime illumination and road network density, whereas hiking is relatively less dependent on lighting conditions. Nighttime illumination does not function merely as a source of brightness; rather, together with vegetation cover, canopy height, terrain slope, and the path network, it shapes exercisers’ overall perceptions of visibility, route legibility, and environmental safety (99, 100). In addition, nighttime illumination exhibits a significant nonlinear relationship with ecological factors. Moderate lighting can improve path recognition and strengthen perceived safety boundaries, whereas tree canopies and vegetation, although beneficial for shade and landscape comfort, may reduce visual permeability when excessively dense. This suggests a synergistic mechanism between nighttime illumination and ecological factors in shaping visual openness and perceived safety (101). Previous research on street runnability likewise shows that jogging is highly sensitive to route quality and perceived safety (53). These findings indicate that the suitability of canal-adjacent spaces for hiking, jogging, and cycling is not determined by any single factor, such as lighting, vegetation, or road networks, but by the interaction of multidimensional environmental elements across different activity types and spatial contexts. As a result, the three activities differ in their threshold effects, spatial distribution, and direction of influence, producing a gradient pattern that declines from the high-value core centered on Beijing-Tianjin toward the periphery, where the high promoting effect zones are dominated by mixed, living, and service spaces. Additionally, other scholars have found that jogging and cycling are more sensitive to water body landscapes and water body density (8, 102), although this was not observed in the present study. Possible explanations include: (1) the excessive diversity of functions and elements along waterfront spaces, coupled with the frequent coexistence of water features alongside variables such as green vegetation and scenic attractions, which may mask their independent effects (9); (2) the abundance of water bodies along canal routes diminishes their marginal appeal to exercisers, whereas in districts with relatively scarce water resources, water features exhibit stronger scarcity effects and significant promotion (103), a phenomenon corroborated by MGWR analysis.

### 5.2 Planning recommendations for physical activity advantage zones along the grand canal

Based on the zoning of advantage zones, the composition of promoting factors, and comparisons of promotion indices, different types of canal-adjacent spaces exhibit significant variation in dominant-factor composition, activity suitability, and spatial conversion efficiency. We therefore propose differentiated planning recommendations, with particular attention to the specific promoting factors, threshold effects, and synergistic relationships identified in the analysis.

#### 5.2.1 Conservation-oriented optimization and walkability enhancement for ecology advantage zones

Ecology advantage zones along the canal corridor are mainly distributed within nature reserves, urban parks and green spaces, and canal corridors in the northern Beijing–Tianjin–Hebei region. The key factors promoting physical activity in these areas are slope gradient, NDVI, and canopy height. Moreover, in zones with strong support for hiking, ecological spaces tend to form concentrated and contiguous clusters, indicating that these ecologically advantageous areas provide particularly strong support for nature-based activities such as hiking (65, 104).

Accordingly, planning in these zones should prioritize protection of the ecological baseline and optimization of the hiking environment, rather than the introduction of high-intensity functional overlays. Through subtle interventions, the continuity of footpaths, the comfort of resting areas, and the legibility of the landscape can be enhanced. Specifically, while maintaining the ecological integrity of these advantage zones, the density of commercial facilities and the intensity of human intervention should be carefully controlled. By incorporating micro-scale landscape nodes, continuous tree-lined paths, ecological revetments, and small resting facilities, planners can strengthen the continuity of blue-green corridors and improve the hiking experience (105). At the same time, the threshold and interaction analyses presented above indicate that although NDVI, canopy height, and slope generally promote hiking, excessively dense vegetation and highly undulating terrain may reduce visual permeability and constrain activities such as jogging and cycling, which depend more strongly on route efficiency and visual continuity (106, 107). Therefore, optimization of ecology advantage zones should balance ecological conservation with visual-corridor organization and path continuity, so as to create waterside ecological spaces that are more suitable for hiking while also accommodating the needs of multiple activity types.

#### 5.2.2 Cultural-tourism vitality stimulation and jogging-support optimization for service advantage zones

Service advantage zones are mainly distributed across urban commercial centers, tourist attraction areas, and office clusters along the middle and lower reaches of the canal. The analysis shows that their key promoting factors include tourist attraction centrality, NDVI, and office complex centrality, indicating that the spatial appeal of these zones is primarily derived from a composite environment that integrates cultural nodes, service functions, and ecological support.

Accordingly, planning for these zones should prioritize the continuity of residents’ daily physical activity, perceived safety, and sense of place, rather than expanding them into specialized competition venues or high-intensity cycling service networks. Specifically, by integrating scenic nodes, waterfront open spaces, and office clusters, planners can strengthen connections between cultural destinations and slow-mobility routes (108), while improving nighttime lighting, wayfinding systems, and rest facilities to enhance the accessibility and legibility of hiking and jogging routes (109). In office-dense areas, priority should be given to improving the connectivity between waterfront jogging tracks and the office-district road network, while supplementing basic support facilities such as washrooms to meet joggers’ needs for continuous routes, environmental safety, and convenient services. At the same time, the findings discussed above indicate that service advantage zones are not the most suitable environments for cycling. In particular, high commercial density and intense nighttime illumination may generate information overload and route-safety concerns for cyclists (110). Therefore, planning in these zones should focus on strengthening the capacity of existing service spaces to support recreational hiking and daily jogging, rather than positioning them primarily as spaces for cycling services (111).

#### 5.2.3 Daily-exercise support and accessibility enhancement for living advantage zones

Living advantage zones are mainly located in urban centers and residential areas of satellite towns. The promoting factors driving these zones are canopy height, accessibility to fitness facilities, and distance to bus stops, indicating that such spaces are particularly suitable for supporting short-distance, low-threshold daily physical activities.

Unlike ecology advantage zones, which prioritize nature-based experiences, and service advantage zones, which emphasize cultural and functional integration, planning for living advantage zones should focus on improving the convenience and continuity of residents’ everyday exercise routines. With accessibility, liveability, and sustained participation as core objectives, stronger connections should be established among fitness facilities, jogging tracks, open green spaces, and public transport stops around residential communities, so as to reduce the time costs and spatial barriers associated with activities such as walking and jogging (112). At the same time, continuity between internal community spaces and surrounding roads should be strengthened, while shade provision, nighttime lighting, and basic wayfinding facilities should be improved to create a safer and more comfortable network for daily activity. The earlier results also indicate that areas favorable for daily cycling often exhibit cross-dimensional promoting factors. Accordingly, optimization of these zones should not be confined to a single dimension, but should instead emphasize the synergistic effects of facility accessibility, ecological quality, and basic supporting services. In addition, living spaces are often embedded within high-density built environments, which can significantly constrain cyclists’ range of movement (113). Planning should therefore address issues such as localized congestion, route discontinuity, and excessive facility concentration, so as to avoid undermining the overall physical-activity experience (106).

#### 5.2.4 Synergistic functional optimization for mixed advantage zones

Mixed advantage zones combine service, living, and certain ecological elements, and rank among the spatial types with the highest physical activity promotion efficiency per unit area. The promoting factors driving physical activity in these zones primarily originate from the service and living dimensions, and cross-dimensional promoting factors and mirrored effects are particularly evident. This suggests that their spatial advantages do not arise from the strengthening of a single function, but rather from the synergistic arrangement and complementarity of multiple elements.

Accordingly, planning for mixed advantage zones should focus on coordinating potential functional conflicts and optimizing integrated support systems. Specifically, based on the threshold and interaction analyses, variables such as nighttime illumination, building density, commercial facility density, and NDVI, which may exert either positive or negative effects depending on the activity type, should be subject to differentiated control, so as to prevent any single factor from becoming overly dominant and undermining overall spatial performance. At the same time, a coordinated package of supportive measures should be implemented by improving public-transport connectivity, optimizing the provision of washrooms and fitness facilities, and enhancing canopy shade as well as the continuity of pedestrian and cycling networks. Because mixed advantage zones are concentrated mainly in the cores of major cities such as Beijing, Tianjin, and Shijiazhuang, and because multi-activity advantage zones are likewise clustered in the core areas of the canal corridor, planning should place greater emphasis on the coordinated organization of high-density mixed-use environments. This would strengthen the capacity of these spaces to accommodate diverse forms of physical activity and, in turn, enhance the overall physical activity promotion potential of canal-adjacent areas.

### 5.3 Implications

**Figure 15:**
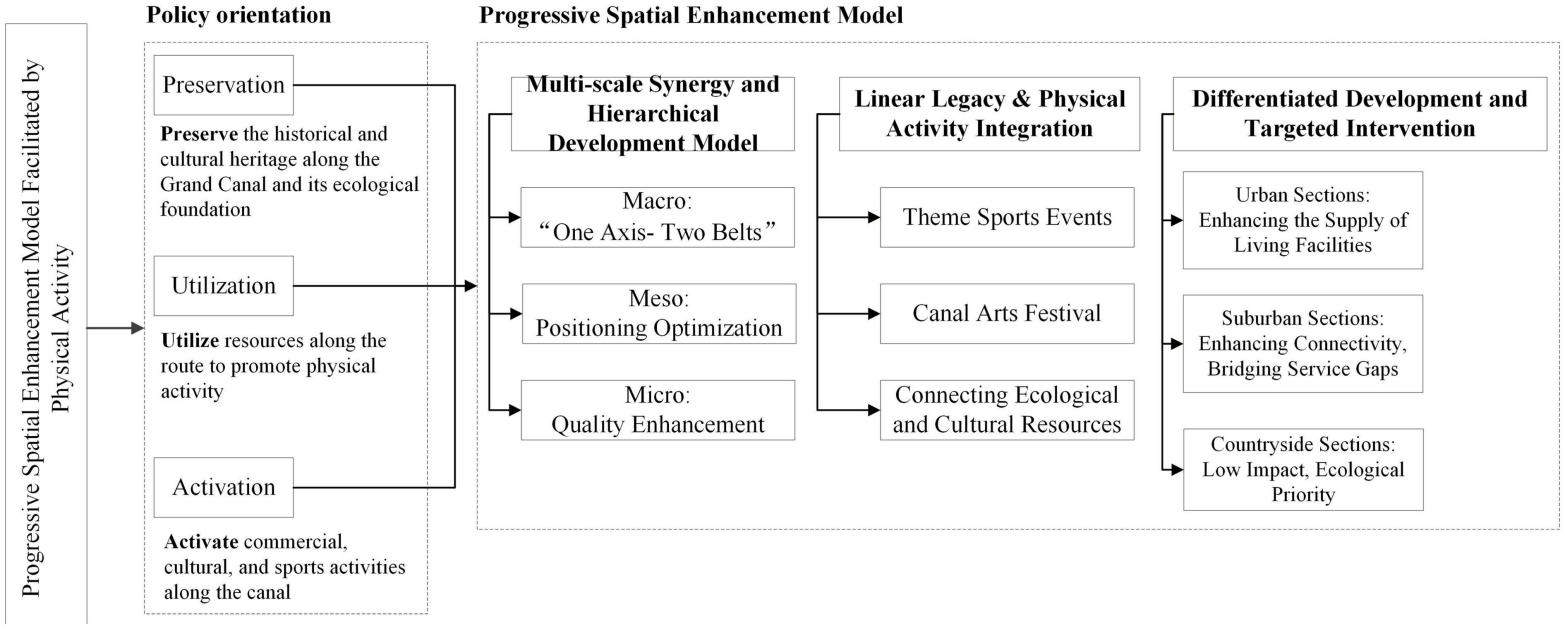
Progressive spatial enhancement model facilitated by physical activity.

This paper provides crucial reference material for planners, designers, and policymakers to understand the role of the Grand Canal in promoting physical activity. We propose a progressive enhancement model for promoting health along the canal corridor (Figure 15). The core principle of this model involves optimizing the canal environment based on local conditions, while preserving historical context and ecological foundations, by integrating the spatial preferences of physical activity participants with the distribution of advantage zones. Facilities are strategically placed within ecological redline boundaries, embedding national fitness objectives into spatial planning to establish a sustainable model that integrates cultural experiences, tourism consumption, and sports recreation.

First, a multiscale and coordinated guidance system should be established to optimize canal-adjacent spatial development along the Grand Canal corridor. Given the pronounced spatial heterogeneity and functional diversity of the Beijing–Tianjin–Hebei section, planning should be advanced at macro, meso, and micro scales. At the macro scale, a general structure of a northern ecological activity belt, central canal core activity axis, and southern living-and-service activity belt could be developed to strengthen regional connectivity and functional differentiation. At the meso scale, differentiated guidance should be implemented according to the identified physical-activity advantage zones. Ecological advantage zones such as Chengde and Zhangjiakou should prioritize conservation-oriented optimization and ecological-corridor continuity; living and service advantage zones should focus on improving slow-mobility networks, facility accessibility, and public-space quality; and mixed advantage zones such as Beijing, Tianjin, and Shijiazhuang should emphasize the coordination of functional conflicts in high-density mixed-use environments. At the micro scale, targeted interventions for footpaths, jogging routes, waterfront nodes, and shaded facilities should be designed according to the key promoting factors and threshold characteristics of different activity types.

Second, heritage conservation should be integrated with the enhancement of daily physical-activity support along the canal corridor. The results show that multi-activity advantage zones are concentrated mainly in the core canal corridor, where relatively dense road networks and mixed-use functions provide favorable conditions for accessibility and route continuity. In urban sections, planning should strengthen links between historic landmarks and the waterfront slow-mobility system, while improving wayfinding, lighting, seating, and washroom provision. In urban–rural transition zones, emphasis should be placed on connecting greenways, villages, orchards, and waterfront open spaces to form continuous activity networks for hiking, jogging, and leisure cycling. In rural sections, ecological and heritage conservation should remain the priority, with strict control over facility density and development intensity to avoid undermining ecological integrity and the natural experience.

Finally, differentiated interventions should be tailored to the dominant promoting factors and local resource conditions of each advantage-zone type. In ecological advantage zones, where slope, vegetation cover, and canopy height are the main promoting factors, planning should prioritize ecological conservation while balancing topography, water bodies, vegetation, and visual corridors to support nature-based activities such as hiking. In living advantage zones, where canopy height, fitness-facility accessibility, and proximity to public transport are more important, planning should focus on improving residential ecological quality, upgrading everyday exercise facilities, and enhancing pedestrian and cycling connectivity. Such targeted and context-sensitive strategies can improve the capacity of canal-adjacent spaces to support active health while advancing the sustainable regeneration of the Grand Canal corridor.

### 5.4 Significance and limitations

Obesity, metabolic disorders, and declining cardiorespiratory fitness resulting from insufficient physical activity have become global health challenges. This paper effectively identifies the interplay between policy orientations, integrating national recommendations for physical activities with linear heritage conservation, and charts an innovative path for integrating culture, commerce, and sports. By combining behavioral trajectory big data with spatial big data, we measured the physical activity promotion effects of canal-adjacent spaces at the regional level. This provides decision-making references for waterfront and linear heritage space planning in the Beijing-Tianjin-Hebei region and potentially in broader areas, while also offering a case study for global nations seeking to implement public health interventions through cultural heritage. This study integrates interpretable machine learning with multiscale spatial analysis and applies this combined framework to public-health research in canal-adjacent spaces along the Grand Canal. It constructs a physical activity promotion index to compare promotion efficiency across different spatial types, thereby providing a quantitative basis for health-oriented planning in linear heritage spaces. At the same time, it offers empirical evidence for research on the public-health-oriented revitalisation of tangible cultural heritage.

This study has several limitations: (1) Although this study collected a large volume of behavioral trajectory data from the Keep platform—primarily capturing linear activities such as walking, jogging, and cycling—the analysis was based on cross-sectional data. It therefore identified only patterns of association and spatial variation between exercise-environment characteristics and physical activity, making it difficult to establish the causal effects of environmental change on activity behavior. Future research could extend the temporal scope of the sample by incorporating multi-year trajectory data and applying causal-inference approaches, such as difference-in-differences, instrumental variables, and structural equation modeling, to strengthen causal identification and improve the robustness and representativeness of the findings. (2) The sample in this study was drawn primarily from active users of the Keep platform and therefore reflects the activity patterns of individuals who proactively record their exercise within a specific period. As such, it does not fully capture the year-round physical-activity patterns of the general population across all age groups, nor does it represent users who do not rely on digital platforms. In addition, because the study period was concentrated in spring, it does not fully account for the influence of seasonal variations in temperature, air quality, and daylight duration on physical activity, which may affect the inclusiveness of the resulting policy recommendations (114). Future research could address this limitation by developing seasonal assessment models or conducting long-term time-series analyses to improve the broader applicability of the findings. (3) This study employs tools such as XGBoost, MGWR, and K-means clustering for factor identification and heterogeneity analysis. Given the rapid iteration of methodologies and technological advancements, future research may incorporate more sophisticated algorithms and frameworks to optimize the technical approach. For instance, integrating spatio-temporal deep learning (LSTM/Transformer, ST-GNN) and graph neural networks could enhance model interpretability and causal explanatory power.

## 6 Conclusion

This study integrates machine learning with spatial econometric models, using the Beijing-Tianjin-Hebei Grand Canal as a case study to reveal the spatial relevances promoting hiking, jogging, and cycling activities. By calculating physical activity promotion indices, it quantifies spatial conversion efficiency and proposes recommendations for optimizing spatial patterns and enhancing activity levels. The study largely confirms prior hypotheses, with key findings as follows: (1) Key promoting factors exhibit widespread nonlinear thresholds and interactive effects. XGBoost and SHAP analyses confirm that spatial factors influence physical activity nonlinearly, exerting promotional effects within specific threshold ranges. (2) Spatial response modes vary significantly across activity types. The same promoting factors exhibit distinct threshold effects and spatial distributions across different physical activities. (3) Cross-dimensional synergy is crucial for enhancing spatial promotion effects. Key promoting factors in specific advantage zones may originate from other dimensions, reflecting the potential for single-function spaces to transition toward multifunctional roles. (4) Physical activity promotion indices along the Grand Canal exhibit a gradient aggregation pattern. Physical activity richness and intensity along the Grand Canal and in large urban agglomerations significantly exceed other regions, forming a gradient structure centered on the Beijing-Tianjin section, with diminishing promotion indices toward the peripheries. (5) Ecology advantage zones exhibit the highest total physical activity promotion, while mixed spaces, despite limited area, demonstrate the highest spatial promotion conversion rate per unit area, particularly in jogging activities. This provides evidence for enhancing functional diversity and experiential richness in canal-adjacent spaces.

## Data Availability

The datasets presented in this study can be found in online repositories. The names of the repository/repositories and accession number(s) can be found in the article/supplementary material.
